# Small Extracellular Vesicles Propagate the Inflammatory Response After Trauma

**DOI:** 10.1002/advs.202102381

**Published:** 2021-10-28

**Authors:** Tanja Seibold, Jonathan Schönfelder, Florian Weeber, André Lechel, Milena Armacki, Mareike Waldenmaier, Christoph Wille, Annette Palmer, Rebecca Halbgebauer, Ebru Karasu, Markus Huber‐Lang, Miriam Kalbitz, Peter Radermacher, Stephan Paschke, Thomas Seufferlein, Tim Eiseler

**Affiliations:** ^1^ Department of Internal Medicine I University Hospital Ulm Albert‐Einstein‐Allee 23 Ulm 89081 Germany; ^2^ Institute of Clinical and Experimental Trauma‐Immunology University Hospital Ulm Albert‐Einstein‐Allee 23 Ulm 89081 Germany; ^3^ Department of Traumatology Hand Plastic and Reconstructive Surgery University Hospital Ulm Albert‐Einstein‐Allee 23 Ulm 89081 Germany; ^4^ Institute of Anesthesiological Pathophysiology and Process Engineering University Hospital Ulm Albert‐Einstein‐Allee 23 Ulm 89081 Germany; ^5^ Department of General and Visceral Surgery University Hospital Albert‐Einstein‐Allee 23 Ulm 89081 Germany

**Keywords:** endothelium, inflammation, neutrophils, small extracellular vesicles, trauma

## Abstract

Trauma is the leading cause of death in individuals under 44 years of age. Thorax trauma (TxT) is strongly associated with trauma‐related death, an unbalanced innate immune response, sepsis, acute respiratory distress syndrome, and multiple organ dysfunction. It is shown that different in vivo traumata, such as TxT or an in vitro polytrauma cytokine cocktail trigger secretion of small extracellular nanovesicles (sEVs) from endothelial cells with pro‐inflammatory cargo. These sEVs transfer transcripts for ICAM‐1, VCAM‐1, E‐selectin, and cytokines to systemically activate the endothelium, facilitate neutrophil‐endothelium interactions, and destabilize barrier integrity. Inhibition of sEV‐release after TxT in mice ameliorates local as well as systemic inflammation, neutrophil infiltration, and distant organ damage in kidneys (acute kidney injury, AKI). Vice versa, injection of TxT‐plasma‐sEVs into healthy animals is sufficient to trigger pulmonary and systemic inflammation as well as AKI. Accordingly, increased sEV concentrations and transfer of similar cargos are observed in polytrauma patients, suggesting a fundamental pathophysiological mechanism.

## Graphical abstract

It is shown that thorax trauma in mice facilitates enhanced release of small extracellular nanovesicles (sEVs) with proinflammatory features, which originate from endothelial cells. Injection of TxT‐plasma‐sEVs into healthy animals triggers local and systemic inflammation. Mechanistically, endothelial‐derived sEVs transfer transcripts for cytokines and adhesion molecules to propagate the inflammatory response. Similar sEVs are detected in polytrauma patients suggesting a fundamental pathophysiological mechanism.

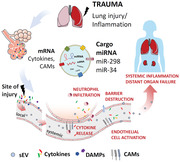

## 1 Introduction

Physical trauma triggers the release of damage‐ and pathogen‐associated molecular patterns (DAMPS/PAMPs),^[^ ^1^ ^]^ neutrophil‐mediated inflammation, cytokine release, and barrier disruption.^[^ ^2^ ^]^ Chest trauma in combination with multiple injuries accounts for up to 25% of all trauma‐related deaths.^[^ ^3^ ^]^ In chest trauma, both lung contusion^[^ ^4^ ^]^ and a marked endotheliopathy determine the posttraumatic response.^[^ ^5^ ^]^ Major complications include acute respiratory distress syndrome (ARDS) and multiple organ dysfunction (MODS)^[^ ^4^, ^6^ ^]^ emphasizing the pivotal role of the lungs for the prognosis of polytrauma patients. The lungs are also targets for secondary inflammatory damage.^[^ ^2^, ^7^ ^]^ Polytrauma, chest trauma, and hemorrhagic shock (HS) can drive a “cytokine storm” with the release of IL1*β*, IL6, CXCL8, and complement factors, resulting in aberrant infiltration of polymorphonuclear cells (PMNs) into lungs,^[^ ^8^ ^]^ highlighting molecular similarities between these conditions. Here, we used a blast‐wave blunt chest trauma mouse model^[^ ^9^ ^]^ to study lung contusion and acute posttraumatic inflammation.^[^ ^10^ ^]^ Recently, small extracellular nanovesicles (sEVs) were identified as crucial mediators of intercellular communication, transducing stress signals, and inflammatory responses,^[^ ^11^ ^]^ for example, during sepsis.^[^ ^12^ ^]^ However, the molecular mechanisms remain unclear and the role of sEVs during posttraumatic inflammation has not been elucidated. Exosomes‐like sEVs are 30–150 nm large membrane‐enclosed nanoparticles of endosomal origin, secreted by almost all cell types, carrying proteins, lipids, DNA, RNA, and miRNAs to reprogram recipient cells.^[^ ^13^ ^]^ Here, we investigate whether trauma quantitatively and qualitatively alters sEV‐release as well as putative functions of sEVs in propagating local and systemic inflammation.

## 2 Results

### 2.1 Different Traumata Result in Increased Serum sEV Concentrations

**Figure 1:**
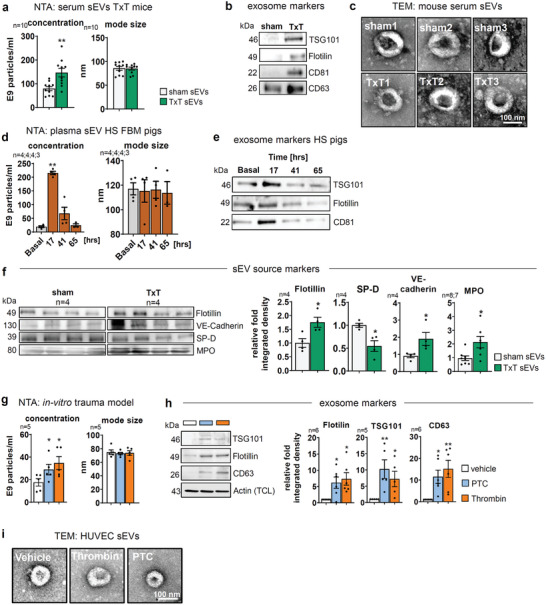
Trauma induces enhanced sEV secretion. a‐c) Murine sEVs isolated from equal amounts of serum of mice 4 h after they were subjected to blast wave‐induced TxT. a) Concentration and mode size of sEVs determined by NTA. b) WB of exosome markers in serum‐sEVs. c) TEM of negative‐stained serum‐sEVs purified from mice. d) Concentration and mode size of sEVs purified from equal amounts of serum from HS FMB‐pigs pre‐ and post‐trauma. e) WB of exosome markers in swine plasma‐sEVs. f) WB of cell‐specific markers in purified sEV preparations from mouse plasma at 4 h after TxT. Graphs show the relative integrated density normalized to the sham control. g) Concentration and mode size of sEVs in the supernatant of HUVECs treated for 24 h with PTC, thrombin, or vehicle. h) Western blot (WB) of exosome markers in PTC, thrombin, or vehicle‐induced sEVs. Actin in TCL was used to control cell numbers. Blots show integrated densities of exosome markers normalized to vehicle. i) Representative TEM images of negative‐stained sEVs from PTC or thrombin‐treated HUVECs. N‐numbers indicate the number of independent samples. Statistical tests: a, f) Two‐tailed unpaired Student's *t*‐test; d, g, h,) RM one‐way ANOVA with Dunnett's multiple comparison test. *P < 0.05; **P < 0.01; ***P < 0.001, ****P < 0.0001; ns: no significant difference.

The secretion of sEVs is altered in response to cellular and molecular stresses.^[^ ^14^ ^]^ We therefore investigated whether different traumatic insults would change serum–sEV concentrations at the peak of acute posttraumatic hyperinflammation. To this end, we have first measured serum sEV concentrations 4 h after a blunt chest trauma in mice (TxT), since a peak in cytokine transcripts was described 3–4 h after lung contusion^[^ ^15^ ^]^ accompanied by PMN infiltration at the site of injury.^[^ ^2^, ^16^ ^]^ Circulating sEVs were isolated by size exclusion chromatography (SEC) ^[^ ^17^ ^]^ from equal serum volumes (**Figure** **1a**; Figure S1a, Supporting information) and subjected to nanoparticle tracking analysis (NTA). Indeed, TxT increased serum‐sEV concentrations by approximately twofold, 4 h after TxT. Isolation of sEVs was further validated following the MISEV criteria.^[^ ^17^, ^18^ ^]^ Exosome markers TSG101, Flotillin, CD81, and CD63 were elevated (Figure 1b) in sEV samples isolated from the serum of TxT mice and transmission electron microscopy (TEM) demonstrated cup‐shaped vesicles whith a size of ≈100 nm (Figure 1c). To investigate whether sEV release would also increase after a different traumatic insult, that is, HS, we have analyzed serum‐sEV concentrations in an familial hypercholesterolaemia Bretoncelles Meishan (FBM) pig HS model.^[^ ^19^ ^]^ Here, sEVs were isolated from venous blood sampled upon induction of anesthesia (base line) as well as 17, 41, and 65 h after resuscitation at the end of a 3 h HS. Again, we detected significantly increased serum‐sEV levels peaking 17 h post‐HS and a steady decrease to almost pre‐shock levels over time (Figure 1d,e). Thus, our data indicate that different traumatic insults facilitate an increase in serum‐sEV concentrations. We therefore went on to elucidate if these sEVs contribute to trauma outcome using the well‐defined TxT mouse model.^[^ ^9^ ^]^ To this end, we initiated a detailed molecular analysis of the respective sEVs in the hyperinflammatory posttraumatic response by first determining the cellular origin of the respective sEVs. Injury patterns following TxT affect lung morphology, but also disturb the endothelial barrier, resulting in edema formation and increased neutrophil infiltration.^[^ ^20^ ^]^ Isolated, purified serum‐sEVs were thus probed with cell‐type‐specific markers for lung atelveolar cells (surfactant protein D, SP‐D), endothelial cells (vascular endothelial cadherin, VE‐cadherin), and neutrophils (myeloperoxidase, MPO) at the 4 h time point after TxT, while total sEV‐load was shown by Flotillin (Figure 1f). Interestingly, VE‐Cadherin and MPO were significantly increased, indicating that serum‐sEVs mostly originated from endothelium and neutrophils, which made us further focus our investigation on sEV‐based communication between these cell types.

### 2.2 Endothelial In Vitro Trauma Model

To study the effect of a traumatic injury on endothelial cells in vitro, we used the barrier‐destabilizing agent thrombin^[^ ^21^ ^]^ and a polytrauma cocktail (PTC) consisting of IL1*β*, IL6, CXCL8, C3a, and C5a‐des‐Arg at concentrations measured in patient serum 24 h after polytrauma to generate a respective trauma microenvironment.^[^ ^22^ ^]^ Treatment of HUVEC monolayers with thrombin significantly destabilized the endothelial barrier with a rapid decrease of transendothelial electrical resistance (TEER) within 20 min. The barrier‐destabilizing effect of PTC was slower in onset, but lasted longer (Figure S1b, Supporting Information). PTC‐treatment of HUVECs for 24 h also significantly impaired expression of CDH5 (VE‐cadherin) and CTNND1 (p120‐catenin), which cooperate in regulating endothelial barrier function.^[^ ^23^ ^]^ Endothelial activation markers ICAM‐1, VCAM‐1, and SELE (CAMs) ^[^ ^24^ ^]^ as well as neutrophil chemoattractant or recruitment factors CXCL8, CXCL5, and CXCL2,^[^ ^25^ ^]^ respectively, were significantly upregulated (Figure S1c,d, Supporting Information). Of note, endothelial cell viability was only slightly impaired (Figure S1e). Interestingly, both PTC‐ and thrombin stimulation also significantly enhanced sEV secretion, as assessed by NTA after 24 h upon purification from HUVEC supernatants by precipitation and SEC (Figure 1g; Figure S1f, Supporting Information). These results were validated by Flotillin, TSG101, and CD63 (Figure 1h). The presence of sEVs was demonstrated by TEM (Figure 1i). We also tested total protein and RNA levels of PTC‐sEVs to evaluate their potential contribution as regulatory cargos. Normalized to particle counts, in particular, total RNA levels were significantly enhanced, whereas total protein was elevated (Figure S1g, Supporting Information). Thus, similar to the in vivo system, endothelial cells respond to an “in‐vitro trauma microenvironment” with a significant increase in sEV secretion. Significantly enhanced levels of RNA cargos further suggest an involvement of this cargo class in the putative regulation of downstream targets.

### 2.3 Endothelial sEV Secretion is Increased by Upregulation of Rab‐GTPases

**Figure 2:**
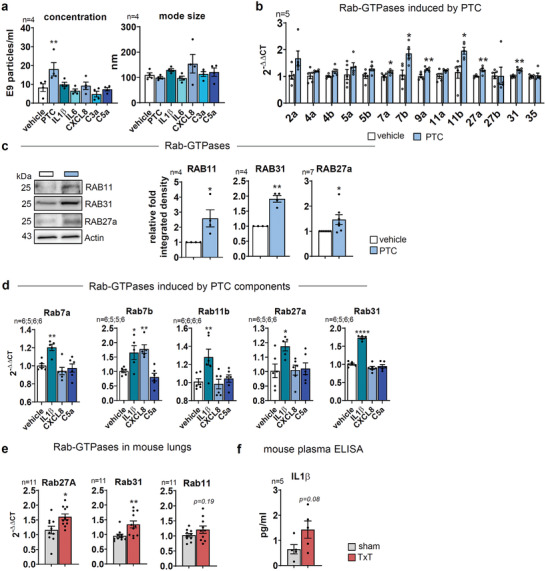
In vitro *trauma* triggers sEV secretion via Rab‐GTPases. a) NTA of HUVEC sEVs after treating the cells for 24 h with vehicle, PTC, and single PTC components. b) qPCR of transcript levels for Rab‐GTPases in HUVECs treated with PTC for 24 h. c) WB of Rab‐GTPases in TCLs of HUVECs after PTC treatment for 24 h. Graphs depict relative integrated densities. d) qPCR of relative transcript levels in HUVECs for RabGTPases after treatment with IL1*β*, CXCL8, and C5a for 24 h. e) qPCR of transcript levels for Rab‐GTPases in lung lysates of TxT mice (4 h). f) Protein levels of IL1*β* in plasma of sham and TxT mice (4 h) assessed by ELISA. N‐numbers indicate the number of independent samples. Statistical tests: b, e, f) Two‐tailed unpaired Student's *t*‐test; c) Ratio paired *t*‐test; a, d) RM one‐way ANOVA with Dunnett's multiple comparison test. *P < 0.05; **P < 0.01; ***P < 0.001, ****P < 0.0001; ns: no significant difference.

Experimental TxT facilitated sEV‐release concomitant with reported local, but also systemic inflammation as indicated by IL1*β*, IL6, or MCP1.^[^ ^7^, ^26^ ^]^ Likewise, PTC increased sEV secretion in vitro. To evaluate a putative contribution of individual inflammatory regulators, HUVECs were treated with respective cytokines and anaphylatoxins. Elevated sEV levels were measured upon treatment with IL1*β*, CXCL8, or C5a, but only PTC‐treatment resulted in a substantial and significant increase in sEV secretion, suggesting an additive effect (**Figure** **2a**). To investigate the underlying molecular mechanism, we determined the expression of Rab‐GTPases involved in exosome‐sEV‐biogenesis. There was a significant upregulation of transcripts for the Rab5‐family member Rab31 (1.24 fold) and Rab11b (1.69 fold) (Figure 2b) upon PTC‐treatment, suggesting increased “endosomal‐sorting‐complexes‐required‐for‐transport”‐(ESCRT)‐independent biogenesis. Moreover, Rab27a expression was significantly enhanced (1.24 fold), indicating increased ESCRT‐dependent sEV‐release.^[^ ^27^ ^]^ Upregulation of these GTPases was also verified on protein level (Rab11: 2.59 fold; Rab31: 1.91 fold; Rab27a: 1.52 fold) (Figure 2c). Besides, elevated Rab7b transcripts (1.75 fold) further point to sorting of more material into multivesicular bodies (MVBs).^[^ ^28^ ^]^

We also tested regulation of Rab‐GTPases by single PTC components, which previously elevated sEV secretion. Like PTC, IL1*β* increased expression of all Rab‐GTPases and thus appears as major driver of sEV‐release in our *in‐vitro* model. CXCL8 only increased the expression of Rab7b. C5a had no significant effect (Figure 2d). *In‐vivo*, Rab27a and Rab31 transcripts were significantly increased in lungs of mice 4hrs after TxT, whereas Rab11b expression was elevated (Figure 2e). This is in line with increased IL1*β* plasma concentrations after TxT (Figure 2f), suggesting that similar biogenesis pathways may be utilized in mice.

### 2.4 Endothelial sEVs Released Upon PTC Treatment Mediate Inflammation and PMN Adhesion In Vitro

**Figure 3:**
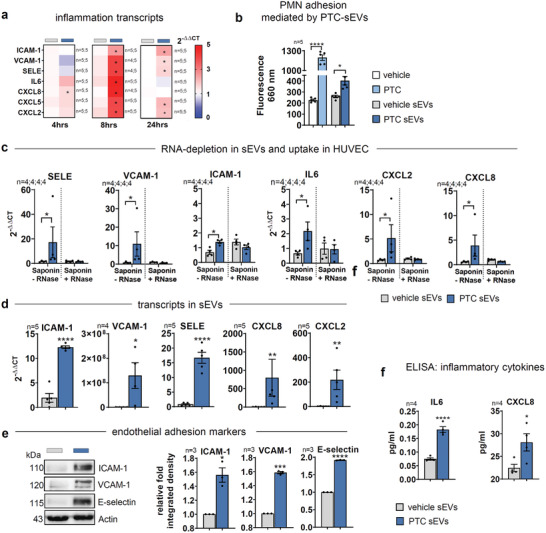
Qualitative changes in sEV cargo after in vitro trauma. a‐c, e‐f, m‐n) HUVECs were treated with vehicle or PTC for 24 h before sEVs were isolated from supernatants. Recipient HUVECs were incubated with respective sEVs. a) qPCR of transcript levels in HUVECs treated with sEVs for 4, 8, and 24 h. Data are shown as heatmap. b) Adhesion of fluorescence‐labeled primary human PMNs to HUVEC cells, which were pretreated with sEVs or PTC for 8 h. c) qPCR of transcript levels in HUVECs after incubation with sEVs for 8 h. sEVs were pretreated with Saponin (0.5%) for permeabilization with or without RNaseA (20 µg mL^–1^) for 10 min. Transcript levels were normalized to the corresponding treatments without sEVs. d) qPCR of transcript levels in sEVs. e) WBs of adhesion molecules in total cell lysates (TCL) of HUVECs treated with sEVs for 8 h. Graphs show relative integrated densities. f) ELISA of CXCL8 and IL6 concentrations in TCLs of HUVECs treated with sEVs for 8 h. N‐numbers indicate independent samples. Statistical tests: a, c, f) Two‐tailed unpaired Student's *t*‐test; e) Ratio paired *t*‐test; d) Mann–Whitney *t*‐test; b) Ordinary one‐way ANOVA with Tukey's multiple comparison test. *P < 0.05; **P < 0.01; ***P < 0.001, ****P < 0.0001; ns: no significant difference.

To identify qualitative changes in sEV cargo that may be associated with local and long‐range systemic effects on the endothelium, we incubated HUVECs for 4, 8 and 24 hrs with sEVs isolated from HUVECs upon vehicle‐ or PTC‐treatment (vehicle‐sEVs, PTC‐sEVs). Equal uptake of sEVs in endothelial cells was verified by uptake experiments in a dose‐dependent manner (Figure S1h). Interestingly, in samples treated with PTC‐sEVs, ICAM‐1, VCAM‐1, SELE, IL6, CXCL8, CXCL2 and CXCL5 transcripts were strongly upregulated after 8 hrs, whereas at the 4 hrs time point only IL6 was significantly increased (**Figure** **3a**). ICAM‐1, VCAM‐1, SELE, CXCL2 and CXCL5 still remained significantly enhanced after 24 hrs.

To control whether soluble contaminates in sEV‐preparations are implicated in the regulation of molecular targets, we additionally subjected HUVEC sEVs isolated after vehicle or PTC‐treatment by precipitation and SEC to ultracentrifugation (100,000g). Subsequently HUVEC cells were incubated with supernatant (SN) and solubilized pellet fractions, respectively. Only the PTC‐sEV pellet fraction, but not the respective SN induced significantly enhanced levels of CAMs and cytokines (Figure S1i, Supporting Information), thus verifying the absence of contaminants in our sEV samples that influence inflammatory responses. Increased expression of adhesion molecules ICAM‐1, VCAM‐1, and E‐selectin is implicated in the inflammatory activation of endothelial cells^[^ ^24^ ^]^ by meditating neutrophil adhesion as well as rolling ball migration.^[^ ^29^ ^]^ IL6 is a general mediator of inflammation,^[^ ^30^ ^]^ whereas CXCL8, CXCL2, and CXCL5 are potent pro‐migratory neutrophil chemokines.^[^ ^25^ ^]^ Thus, we tested the role of PTC‐sEVs in the endothelial adhesion of PMNs. The binding of PMNs was facilitated by PTC‐sEVs after 8 hrs (Figure 3b) and adhesion was increased in a dose‐dependent manner (Figure S1j, Supporting Information). To determine whether functional mRNA transfer by sEVs was involved in the regulation of targets, sEVs were permeabilized with saponin and treated with RNase before incubation with HUVECs, demonstrating that effects of sEVs on cellular transcript levels for CAMs as well as cytokines were now abolished (Figure 3c). We further detected mRNAs for CAMs, CXCL8, and CXCL2 by qPCR directly in sEVs (Figure 3d). To validate that sEV‐derived transcripts are indeed intact, we additionally performed exemplary in vitro translation experiments with total RNA isolated from vehicle‐ and PTC‐sEVs. WB analysis of synthesized proteins demonstrated that ICAM‐1, IL8, and GAPDH control transcripts were generated at the proper molecular weights and detected by specific monoclonal antibodies, pointing to functional, full‐length mRNA molecules (Figure S1k, Supporting Information). Moreover, we detected significantly increased protein levels for CAMs in lysates of HUVECs treated with PTC‐sEVs (Figure 3e) as well as production of IL6 and CXCL8 after 8 h (Figure 3f).

Several RNA‐binding proteins (RBPs) were previously described to load mRNA and miRNAs into sEVs. To identify relevant co‐factors, we went on to subject vehicle‐ and PTC‐sEVs to mass spectrometry (MS). To control the quality of the MS data, intensities of proteins annotated as extracellular vesicles (GO:1 903 561) within the Uniprot database (www.uniprot.org<http://www.uniprot.org>) were compared to total protein intensities in the respective samples and EV‐annotated hits were responsible for 69.2% of total protein intensities (see Supporting Information Data File 1). We have also cross‐referenced the list of significant, differentially regulated proteins to the GO 2021 cellular components repository with the terms “cytoplasm” and “extracellular vesicular exosome,” where an overlap of 47.6% and 73.5% was detected, respectively, again indicating a profound enrichment of sEV‐related signatures (Figure S2a, Supporting Information). Moreover, of the 795 identified proteins, 763 were associated with the term “exosomes” in the Vesiclepedia database (9791 entries) and all 179 significantly regulated targets were part of this group (Figure S2b, Supporting Information).

**Figure 4:**
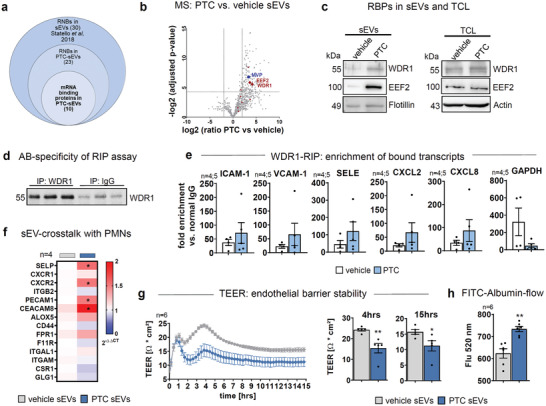
Packaging of transcripts in sEVs by RNA binding proteins. a) Comparison of RBPs identified in this study by MS and Statello et al. b) Volcano blot of differentially regulated proteins in PTC versus vehicle sEVs identified by MS. Enriched mRNA‐RBPs are highlighted in red, miRNA‐RBPs in blue. c) RBP expression in sEVs and TCLs of vehicle and PTC treated cells for 24 h. One of three independent experiments is shown. d) WB of WDR1 in lysates after RIP assays to validate antibody specificity. e) Fold enrichment of WDR associated transcripts in RIP assays normalized to IgG CT‐values. f) qPCR of neutrophil adhesion‐relevant transcripts in human PMNs after treatment with sEVs for 8 h derived from HUVECs at the indicated conditions. g) TEER kinetic in HUVEC monolayers on transwell filters treated with PTC‐sEVs. h) FITC‐Albumin flow across HUVEC monolayers on transwell filters after 8 h. N‐numbers indicate independent samples. Statistical tests: f, g, h) Two‐tailed unpaired Student's *t*‐test; *P < 0.05; **P < 0.01; ***P < 0.001, ****P < 0.0001; ns: no significant difference.

A subsequent analysis for RBPs in our MS data indicated 23 hits of which 10 are known to bind mRNA^[^ ^31^ ^]^ among the group of significantly upregulated protein (**Figure** **4a**; Supporting Information Data File 1). The top two upregulated RBPs, WD‐repeat‐containing‐protein‐1 (WDR1) and EEF2, vital factors for actin‐regulation and transcription, respectively,^[^ ^31^ ^]^ were enhanced in PTC‐sEVs (Figure 4b,c), but not differentially expressed in cell lysates (Figure 4c). Using RNA‐immunoprecipitation (RIP), we then demonstrated increased binding of inflammatory CAMs and CXCL2/CXCL8 transcripts to WDR1‐RIPs of PTC‐treated cells, when compared to vehicle controls. Contrary, GAPDH was not enriched but rather depleted in RIPs of PTC‐treated cells, effectively acting as a control for transcripts that are not associated with an inflammatory phenotype (Figure 4d,e).

To further evaluate a putative education of primary PMNs by endothelial‐derived sEVs, we have also interrogated PMN‐chemoattractant receptors and adhesion factors by qPCR. Indeed, CXCR2 (CXCL8‐receptor) and PECAM‐1 transcripts, that regulate the interaction of PMNs with the endothelium, in particular during diapedesis, were significantly increased 4 h after incubation with PTC‐sEVs (Figure 4f). Additionally, CEACAM8 and SELP that facilitate endothelial‐neutrophil interactions were significantly upregulated^[^ ^32^ ^]^ (Figure 4f).

In summary, these data indicate that PTC‐sEVs prepare the endothelium for augmented interaction with neutrophils, but also regulate complementary targets in PMNs.

### 2.5 sEVs Released from Endothelial Cells Upon PTC Treatment Disrupt The Endothelial Barrier

**Figure 5:**
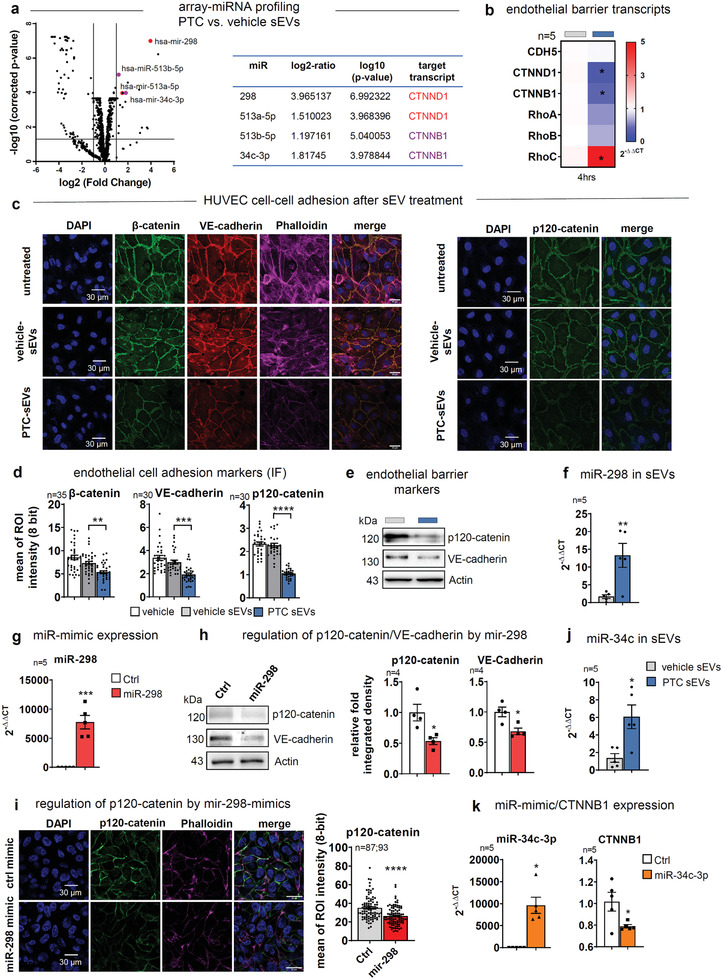
miRNAs mediate barrier breakdown after treatment with PTC‐sEVs. a) Volcano blot of differentially regulated miRNAs isolated from vehicle‐ and PTC‐sEVs analyzed by a miRNA micorarray. b) qPCR of relative transcript levels for barrier‐relevant targets in HUVEC cells treated with sEVs for 4 h. Data are shown as heatmap. N‐numbers indicate the number of independent samples. c) Confluent HUVEC monolayers treated with vehicle and PTC‐sEVs for 8 h. Barrier disruption was assessed by quantitative mean of ROI analysis at AJs of confocal image sections stained with VE‐cadherin, *β*‐catenin, Phalloidin, and DAPI or p120‐catenin and DAPI, respectively, recorded with similar settings. Scale: 30 µm. d) Statistical analysis of mean of ROI intensities measured at AJs in (c), with three‐replica ROIs per junction, *n* = 30 junctions. e) WB of VE‐cadherin and p120‐catenin in TCL of HUVECs treated with vehicle or PTC‐sEVs for 24 h. f) qPCR for relative expression of miR‐298 in sEVs of HUVECs treated with PTC and vehicle for 24 h. g) qPCR for relative expression of mir‐298 24 h after transfection of miR‐298 mimics in HUVECs. h) WB of the mir‐298 target p120‐catenin and VE‐cadherin in HUVECs after transfection with mir‐298 mimics. Graphs depict relative integrated densities. i) Confluent HUVEC monolayers were transfected with mir‐298 mimics and incubated for 48 h. Depletion of p120‐catenin was assessed by quantitative mean of ROI analysis at AJs of confocal image sections stained with p120‐catenin, Phalloidin, and DAPI, recorded with equal settings. Sale: 30 µm. The graph displays the statistical analysis of the mean of ROI intensities measured at AJs, with five‐replica ROIs per junction, *n* = 87, 93 junctions. j) qPCR for relative expression of miR‐34c‐3p 24 h after treatment of HUVECs with PTC‐sEVs. k) qPCR to quantify miR‐34c‐3p and CTNNB1 transcripts levels after transfection with miR‐34c‐3p mimics. Statistical tests: b, f, g, i, j, k) Two‐tailed unpaired Student's *t*‐test; h) Ratio paired *t*‐test; d) Ordinary one‐way ANOVA with Tukey's multiple comparison test. *P < 0.05; **P < 0.01; ***P < 0.001, ****P < 0.0001; ns: no significant difference.

Next, we investigated if PTC‐sEVs would regulate endothelial barrier stability. Disruption of the endothelial barrier is a critical pathophysiological factor, enhancing the passage of neutrophils toward the site of injury.^[^ ^33^ ^]^ Indeed, PTC‐sEVs decreased TEER with a maximum of ≈4 h. The barrier remained significantly disturbed up to the end of the measurement period (15 h; Figure 4g). Barrier failure was validated by a significant increase in FITC‐albumin‐flow after 8 h (Figure 4h). Endothelial barrier stability crucially relies on the availability of VE‐cadherin‐mediated contacts at the plasma membrane.^[^ ^34^ ^]^ In this context, CTNND1 (p120‐catenin) is required for stabilizing VE‐cadherin adhesion complexes.^[^ ^23^ ^]^ Moreover, *β*−catenin (CTNNB1) is known to connect VE‐cadherin to several actin‐binding proteins and thus F‐actin.^[^ ^35^ ^]^ Since miRNAs, which could also down‐regulate these barrier‐relevant factors, are major sEV cargos, we analyzed the miRNA content of EVs from PTC‐ and vehicle‐treated HUVECs using a microarray (Supporting Information Data File 2). Indeed, 19 miRNAs were significantly upregulated (log_2_‐ratio ≥ 1) and 54 downregulated in PTC‐sEVs (log_2_‐ratio ≤ ‐1; **Figure** **5a**). Among the top upregulated miRNAs, we identified hsa‐mir‐298 that was previously demonstrated to impair CTNND1 expression.^[^ ^36^ ^]^ Moreover, the much weaker expressed mir‐513a‐5p was suggested to regulate CTNND1 expression in mirTarBase (mirtarbase.mbc.nctu.edu.tw). MirTarBase analyses also indicated the miR‐34 family is able to regulate the expression of CTNNB1.^[^ ^37^ ^]^ Here, we identified mir‐34c‐3p and additionally mir‐513b‐5p as CTTNB1 targeting miRNAs (Figure 5a). A downregulation of CTNND1 and CTNNB1 transcripts was validated by incubating HUVECs with PTC‐sEVs for 4 h (Figure 5b). Endothelial barrier function is also critically impacted by Rho‐GTPase (RhoA, B, C).^[^ ^38^ ^]^ To this end, we found an increased RhoC expression, which likely aids in destabilizing VE‐cadherin adhesion complexes by PTC‐sEVs (Figure 5b). Downregulation of p120‐ and *β*‐catenin at adherens junctions (AJ) was further corroborated using immunofluorescence (IF) in HUVEC monolayers after 8 h. In line with reduced p120‐catenin levels, VE‐cadherin and *β*‐catenin expression^[^ ^23^ ^]^ was significantly impaired (Figure 5c,d). This phenotype was also validated at 24 h in TCL (Figure 5e). Moreover, enhanced levels of hsa‐mir‐298 and hsa‐mir‐34c‐3p were detected in PTC‐sEVs by qPCR (Figure 5f,j). Besides, mir‐298 mimics significantly downregulated p120‐catenin at 24 and 48 h (Figure 5g‐i), which also translated into a reduction of VE‐cadherin expression (Figure 5h), while hsa‐mir‐34c‐3p mimics significantly impaired expression of CTNNB1 transcripts (Figure 5k). In summary, given the prominent upregulation of mir‐298 in PTC‐sEVs, CTNND1 is likely the major regulator of endothelial barrier stability, but may be aided by the other miRNAs as well as RhoC. miRNAs are packaged into MVBs by dedicated RBPs. Interestingly, we detected enhanced levels of the RBP major vault protein (MVP) in PTC‐sEVs (Figure 4b; Figure S2c, Supporting Information). MVP was previously described to transfer different miRNAs into sEVs.^[^ ^39^ ^]^ Thus, we suggest MVP could be utilized for packaging the respective miRNAs during PTC stimulation.

### 2.6 Role of sEVs in Transduction of Inflammation and Posttraumatic Response

**Figure 6:**
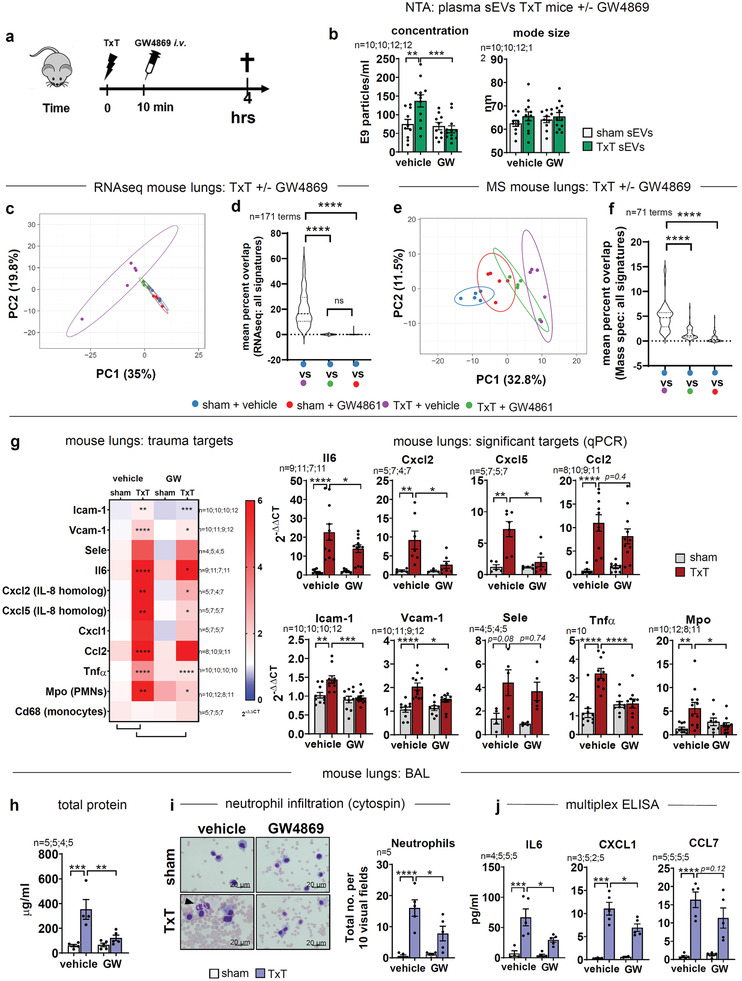
Inhibition of sEV‐biogenesis by GW4869 reverses TxT local and systemic molecular phenotypes. a) Scheme of TxT experiments in mice. Groups: sham+vehicle, sham+GW4869, TxT+vehicle, TxT+GW4869. Animals were sacrificed 4 h after TxT. b) NTA of sEVs purified from equal volumes of mouse plasma. c) PCA with all differentially regulated transcripts (log_2_ ≥ +2, ≤ ‐2) assed by RNAseq of mouse lungs. d) Mean percent overlap for trauma‐associated enriched terms in differentially regulated transcripts for indicated groups. Terms were identified from differentially regulated transcripts in TxT+vehicle versus sham+vehicle conditions by unweighted EnrichR meta‐analysis (log_2_ ≥ +2, ≤‐2; first 20 significant enriched terms selected). e) PCA of all significantly regulated proteins (log_2_ ≥+1, ≤‐1) assessed by MS of mouse lungs. f) Mean percent overlap for trauma‐associated enriched terms in the indicated groups. Terms were identified for TxT+vehicle versus sham+vehicle conditions by unweighted EnrichR meta‐analysis of all significantly up‐ or downregulated proteins. g) qPCR of transcripts for inflammation‐ and barrier‐relevant genes in mouse lungs. Expression was normalized to sham+vehicle. h) Total protein concentration in BAL of mice. i) Differential staining of BAL‐resident cells on cytospin slides. The arrowhead indicates neutrophils. The graph depicts neutrophil counts in ten random visual fields/slide. j) Multiplex‐ELISA of indicated cytokines in BAL‐fluid. Unless stated otherwise N‐numbers indicate independent samples. Statistical tests: b, g, h, i, j,) Ordinary one‐way ANOVA with Holm‐Sidak multiple comparison test. g, j) Outliers were removed based on a ROUT outlier test (Q = 5%). *P < 0.05; **P < 0.01; ***P < 0.001, ****P < 0.0001; ns: no significant difference.

So far we demonstrated that TxT increased sEV‐release in mice and we identified molecular targets in vitro. We therefore asked next, whether sEVs secreted upon TxT also affect the posttraumatic response in vivo. To this end, mice were injected with an exosome‐sEV‐biogenesis inhibitor: GW4869^[^ ^40^ ^]^ 10–15 min after TxT (**Figure** **6a**). NTA demonstrated significantly enhanced sEV concentrations 4 h after the insult, which were reverted by GW4869 (Figure 6b). To investigate the impact of GW4869 on TxT outcome in lungs, we have analyzed changes in the whole transcriptome and proteome (Supporting Information Data File 3–8). Numbers indicating significantly regulated genes during RNAseq are listed in Figure S2d, Supporting Information. In conclusion, significantly regulated transcripts after TxT were strongly reduced by GW4869. Principal component analysis (PCA) demonstrated clearly separated clustering of TxT from TxT (+GW4869) samples, which were also overlapping with sham (+GW4869) and sham (+vehicle) conditions in a PC1/PC2 blot (Figure 6c). Using unweighted EnrichR meta‐analysis,^[^ ^41^ ^]^ we identified 171 significantly enriched trauma‐associated terms amongst the first 20 enriched signatures in TxT (+vehicle) versus sham (+vehicle) samples. For GW4869 conditions these signatures were abrogated (Figure 6d; Figure S2d, Supporting Information). Terms were further sorted into subgroups according to physiological or molecular parameters (Figure S2e, Supporting Information). We also identified terms associated with exosomes in Jensen's Compartment database (Figure S2f, Supporting Information). Again, percent overlap was almost completely lost in GW4869‐treated samples. In addition, we used MS to identify changes in protein expression. Numbers for significantly regulated proteins in MS are listed in Figure S3a, Supporting Information. PCA analysis demonstrated a separation of GW4869‐treated TxT samples from TxT (+vehicle) conditions into the direction of the sham cluster (Figure 6e). Unweighted EnrichR meta‐analysis for upregulated proteins in TxT (+vehicle) versus sham (+vehicle) samples identified 26 significantly enriched terms sorted in the groups: neutrophil immunity, apoptosis, and endothelial barrier function (Figure S3a and S3b, Supporting Information), whereas downregulated proteins resulted in 45 enriched terms mainly involved in transcription and protein synthesis. Accordingly, treatment of TxT mice with GW4869 significantly reduced the percent overlap for all terms, as well as subgroups (Figure 6f; Figure S3b, Supporting Information). Again, signatures for exosomes were significantly enriched in TxT and less enriched in TxT/vehicle + GW4869 conditions (Figure S3b, Supporting Information). Thus, transcriptome and protein profiling from lungs upon TxT with the concomitant intervention of GW4869 suggest “trauma‐sEVs” are vital regulators of inflammation, barrier destabilization, and neutrophil‐mediated immunity. To verify normalization of inflammation by GW4869, we also detected relevant transcripts by qPCR in lungs: Il6, Cxcl2, and Cxcl5 (mouse CXCL8 homologs),^[^ ^42^ ^]^ the chemoattractant Cxcl1, known to bind neutrophils to inflammatory sites^[^ ^43^ ^]^ and Ccl2, which mediates monocyte and neutrophil chemoattraction^[^ ^44^ ^]^ as well as TNF‐*α*, described to modulate neutrophil diapedesis^[^ ^45^ ^]^ were evaluated. The presence of neutrophils in the lungs was shown by Mpo,^[^ ^46^ ^]^ while monocytes were identified by Cd68.^[^ ^47^ ^]^ Endothelial activation was evaluated by CAM expression (Figure 6g). Indeed, Il6, Cxcl‐2/5, Ccl2 as well as Tnf*α* and Mpo were significantly upregulated in lungs 4 h after TxT, whereas expression was significantly reverted by GW4869, in line with MS. Of note, monocytes (Cd68) were not significantly affected. Similar to the in vitro trauma model, endothelial activation was demonstrated by the increase in CAM transcripts and reversed by GW4869. Additionally, multiplex‐ELISAs of lung lysates after TxT showed enhanced levels of IL6 and the PMN chemoattractants CXCL1, CCL2 as well as CCL7^[^ ^43^, ^48^ ^]^ that were significantly reduced by GW4869 treatment (Figure S3c, Supporting Information). Inflammation and barrier breakdown were analyzed using bronchoalveolar lavage (BAL). Total BAL‐protein, indicating barrier leakage, was significantly increased after TxT, and this effect was again diminished by GW4869 injection (Figure 6h). PMN infiltration after TxT was quantified using BAL‐cytospins. GW4869 treatment significantly decreased PMN counts (Figure 6i). Regulation of pro‐inflammatory cytokines in BAL fluids is shown for IL6, CXCL1 as well as CCL7, in line with the literature^[^ ^48^ ^]^ (Figure 6j).

**Figure 7:**
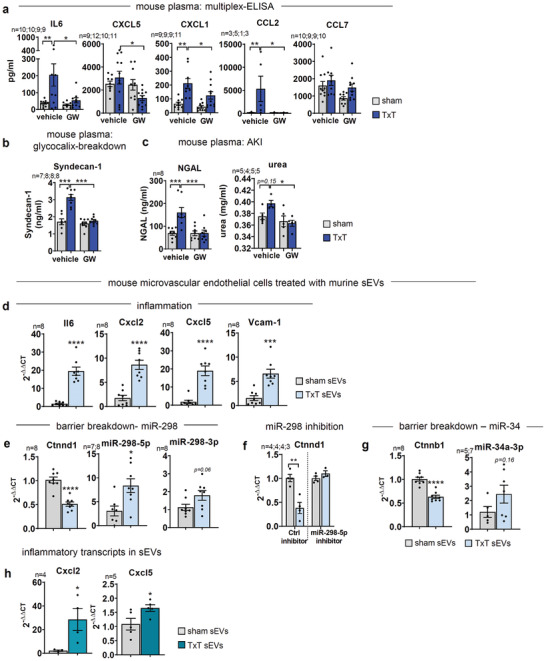
Systemic inflammation and transfer of transcripts to endothelial cells by murine plasma‐sEVs after TxT. a) Multiplex‐ELISA of selected cytokines in the plasma of TxT and sham mice. All analyzed cytokines are shown in Figure S4a, Supporting Information. b) Glycocalyx breakdown assessed by Syndecan‐1‐ELISA in mouse plasma. c) Early AKI assessed by NGAL‐ELISA and urea measurements in mouse plasma. d‐g) Mouse primary microvascular lung endothelial cells were treated with purified sEVs from equal plasma volumes of TxT and sham mice for 8 h and transcripts evaluated by qPCR. d) Expression of inflammation‐relevant genes after treatment with sham‐ and TxT‐sEVs. e) Expression of barrier‐relevant Ctnnd1 and targeting miRNAs mmu‐mir‐298‐5p/3‐p quantified by qPCR induced by sham‐ or TxT‐sEVs. f) Expression of Ctnnd1 after transfection of endothelial cells with miR‐298‐5p inhibitor before treatment with the indicated sEVs. Transcript levels were normalized to the corresponding sham‐sEV treated samples. g) Expression of barrier‐relevant Ctnnb1 and targeting miRNA mmu‐mir‐34a‐3p quantified by qPCR. h) Transcript levels of Cxcl2 and Cxcl5 detected by qPCR in TxT‐ and sham‐sEVs. N‐numbers indicate independent samples. Statistical tests: a) Ordinary one‐way ANOVA with Holm‐Sidak multiple comparison test. a, b) Outliers were removed based on the ROUT outlier test (Q = 5%); d, e, f, g, h) Two‐tailed unpaired Student's *t*‐test. *P < 0.05; **P < 0.01; ***P < 0.001, ****P < 0.0001; ns: no significant difference.

To explore the consequence of these data on systemic inflammation in response to physical trauma, we analyzed plasma cytokine levels after TxT by multiplex‐ELISA (**Figure** **7a**; Figure S4a, Supporting Information, and Supporting Information Data File 9). IL6 was significantly upregulated 4 h after trauma and levels were significantly reduced by GW4869. Likewise, chemokines CXCL1, CXCL5, and CCL2 were significantly increased and again dampened by GW4869 treatment. We also evaluated the status of the endothelial barrier after TxT or GW4869‐injection, quantifying glycocalyx breakdown using plasma‐Syndecan‐1 (Figure 7b). ^[^ ^49^ ^]^ Concurrent regulation of distant, acute kidney injury (AKI) was assessed by the plasma‐markers NGAL^[^ ^50^ ^]^ and the clinical parameter urea (Figure 7c). GW4869 prevented both, increases in Syndecan‐1 as well as plasma‐NGAL and urea levels upon TxT. Thus, GW4869 treatment 10–15 min after TxT substantially prevented sEV‐dependent reprogramming of the endothelium and ameliorated local as well as systemic inflammation.

### 2.7 Molecular Regulators are Transferred via TxT‐Plasma‐sEVs

To further validate the molecular targets, previously identified in the sEVs of the in vitro trauma model, we have treated mouse primary lung microvascular endothelial cells with sEVs of TxT and sham mice for 8 h. Again, increased expression of Il6, Cxcl2/Cxcl5, and the exemplary endothelial adhesion marker Vcam‐1 was detected upon TxT‐sEV treatment using qPCR (Figure 7d). Endothelial barrier dysfunction was indicated by downregulation of Ctnnd1 as well as increased expression of mmu‐mir‐298‐5p and ‐3p (Figure 7e).^[^ ^36^ ^]^ To this end, TargetScan7.1‐analysis displayed ten binding sites in the 3’‐UTR of mouse Ctnnd1 for mmu‐mir‐298‐5p, whereas one binding‐site was described for mmu‐mir‐298‐3p. To demonstrate the direct inhibition of Ctnnd1 by sEV‐resident mir‐298, the main regulatory miRNA found in our in vitro studies, we utilized a mir‐298‐5p inhibitor to reverse downregulation of Ctnnd1 upon TxT. Indeed, we were able to demonstrate a direct involvement of TxT‐sEV and mir‐298‐5p in the murine system (Figure 7f). In addition, we show downregulation of Ctnnb1 and transfer of the mir‐34 family member mmu‐mir‐34a‐3p, which could target *β*‐catenin^[^ ^51^ ^]^ in TxT‐sEV treated samples (Figure 7g) as well as upregulation of the most prominent transcripts Cxcl2 and Cxcl5 directly in TxT‐sEVs (Figure 7h). Thus, our data indicate similar cargos are increased in mouse and the in vitro “trauma‐sEVs.”

### 2.8 Injected TxT‐sEVs Facilitate Systemic, Pulmonary, and Renal Inflammation

**Figure 8:**
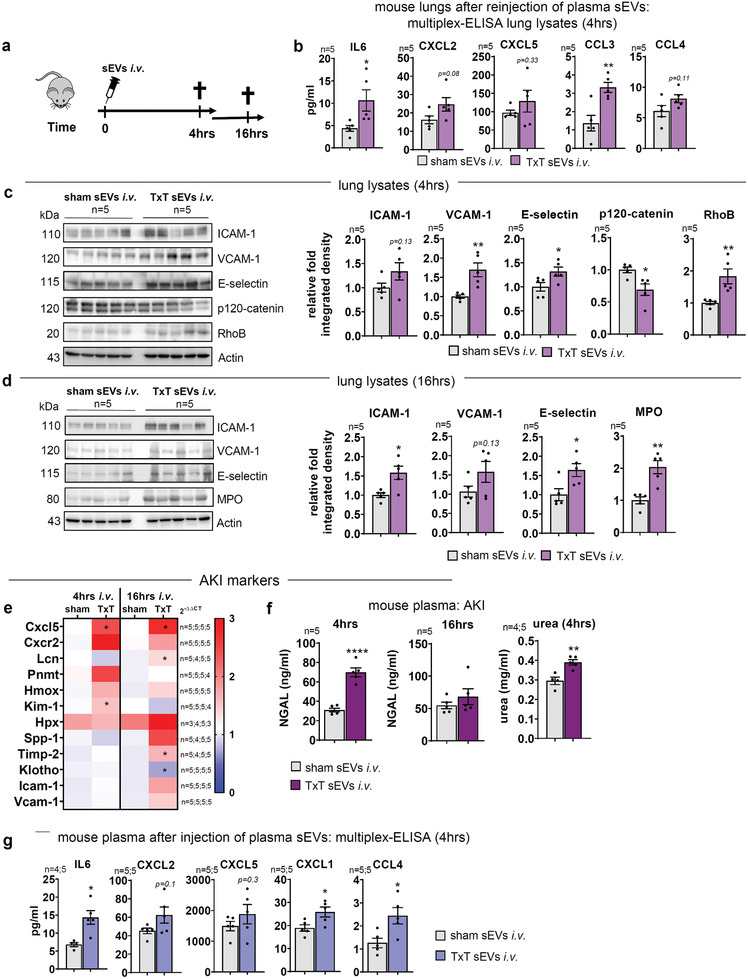
Injection of TxT‐sEVs into naïve mice propagates endothelial activation, inflammation, and organ damage. a) Scheme for injection experiments. Mice were sacrificed 4 or 16 h after the injection of sEVs. () Multiplex‐ELISA of the indicated cytokines in lung lysates of injected mice after 4 h. c) WBs of CAMs, p120‐catenin, and RhoB in total lung lysates of mice 4 h after sEV‐injection. Graphs depict relative integrated densities. d) WB of CAMs, MPO, and ß‐catenin in total lung lysates of mice 16 h after sEV‐injection. Graphs depict relative integrated densities. e) qPCR of AKI markers in total kidney lysates 4 and 16 h after sEV injection. mRNA levels were normalized to sham animals at corresponding time points. Data are shown as heatmap. f) NGAL‐ELISA in mouse plasma after 4 and 16 h and urea determined after 4 h. g) Multiplex‐ELISA of the indicated cytokines in mouse plasma 4 h after sEV‐injection. N‐numbers indicate independent samples. Statistical test: b, c, d, e, f, g) Two‐tailed unpaired Student's *t*‐test. *P < 0.05; **P < 0.01; ***P < 0.001, ****P < 0.0001; ns: no significant difference.

The exosome‐sEV‐biogenesis inhibitor GW4869 has been previously used in several studies to inhibit sEV release in vitro and in vivo.^[^ ^40^, ^52^ ^]^ Nevertheless, given its role as an inhibitor of nSMAse‐mediated ceramide synthesis during sEV‐biogenesis and some anti‐inflammatory properties,^[^ ^52^, ^53^ ^]^ we wanted to further substantiate the role for TxT‐sEVs in mediating systemic inflammatory processes. Thus, 50% of TxT and sham plasma‐sEVs were isolated, purified, and injected into healthy C57BL/6 animals. Lungs and kidneys were analyzed 4 and 16 h after sEV injection (**Figure** **8a**). Indeed, after 4 h we already detected significant upregulation of IL6 and increase of CXCL2/CXCL5 in the lungs (Figure 8b). In addition, the neutrophil chemoattractant CCL3^[^ ^54^ ^]^ was significantly enhanced and increased CCL4 levels pointed to an amplified vascular inflammatory response.^[^ ^55^ ^]^ Besides CAMs were increased in lung lysates 4 and 16 h after injection (Figure 8c,d). Moreover, p120‐catenin levels were significantly impaired, while RhoB expression was enhanced (Figure 8c), pointing to a destabilization of the endothelial barrier after 4 h^[^ ^23^, ^38^ ^]^ and increased PMN infiltration as indicated by MPO after 16 h (Figure 8d). These data were validated by quantifying transcripts for CAMs, chemokines Cxcl2/5, RhoA/B/C, Mpo, and the lung damage marker Scgb1a (Cc16)^[^ ^56^ ^]^ 4 and 16 h after injection (Figure S4b, Supporting Information). After 4 h, Cxcl2, Vcam‐1 and Scgb1a transcripts were significantly elevated, whereas expression of the other regulators, except for RhoC, was non‐significantly increased. We further observed enhanced Mpo levels after 4 h and significantly higher levels after 16 h.

To substantiate our data on the involvement of TxT‐sEVs in distant organ damage, we interrogated published AKI‐relevant transcripts.^[^ ^57^ ^]^ Kim1 was significantly upregulated by TxT‐sEVs after 4 h, whereas Lcn2 (NGAL) and Timp2 were upregulated 16 h after administration (Figure 8e). In turn, Klotho was significantly downregulated at 16 h, in line with the literature.^[^ ^57^ ^]^ Thus, five of nine markers were significantly changed, while additional transcripts were elevated (Figure 8e), suggesting an effect of sEVs on AKI. Besides, increased levels of Icam‐1 and Vcam‐1 as well as Cxcl5 further point to endothelial activation after 16 h. These data were corroborated by plasma NGAL‐ELISAs and urea measurements demonstrating AKI 4 h after TxT‐sEV injection (Figure 8f). A systemic inflammatory response was indicated by increased plasma levels of IL6, CXCL2/CXCL5, CXCL1 as well as CCL4, further pointing to amplified vascular inflammation 4 h after sEV‐treatment (Figure 8g).^[^ ^55^ ^]^ In summary, our data show injected TxT‐sEVs drive systemic inflammation.

### 2.9 Increased Plasma‐sEVs in Polytrauma Patients Propagate Endothelial Inflammation

**Figure 9:**
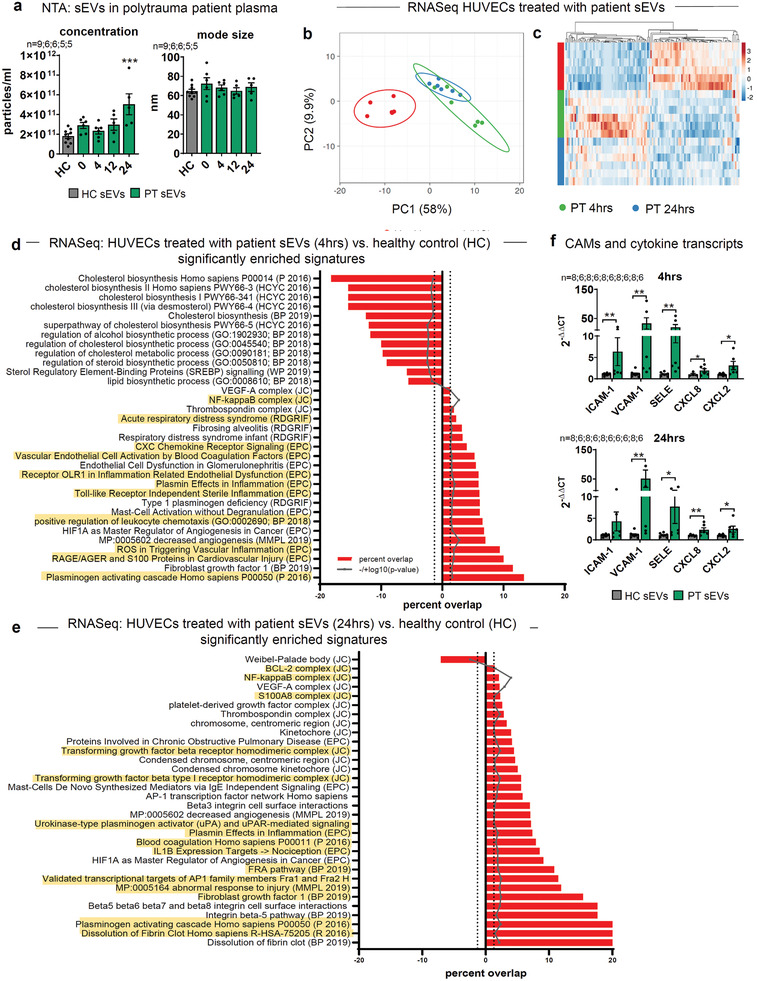
Plasma‐sEVs from polytrauma patients facilitate gene expression associated with inflammation and neutrophil immunity. a) NTA of sEVs isolated from 100 µL plasma of healthy controls (HC) or PT‐patients upon admittance to the ER (T0) and 4, 12, and 24 h after trauma. Particle concentrations were normalized to total plasma protein. b) PCA analysis of all significantly regulated genes detected by RNAseq in HUVECs after 8 h‐treatment with plasma‐sEVs from HC and PT‐patients (isolated 4 h and 24 h after trauma). c) Unsupervised clustering of all significantly regulated genes used in (b). d) Enriched trauma‐associated terms found in HUVECs treated with sEVs from PT‐patients (4 h after trauma) versus HC, expression cut‐off: log_2_ ≥ +0.135, ≤‐0.135. The graph depicts percent overlap for significantly enriched terms and ±log10 (*P*‐values). e) Enriched trauma‐associated terms found in HUVECs treated with sEVs from PT‐patients (24 h after trauma) versus HC, expression cut‐off: log_2_ ≥ +0.135, ≤ ‐0.135. The graph depicts percent overlap for significantly enriched terms and ±log10 (*P*‐values). f) qPCR of identified endothelial adhesion markers and cytokines after treatment of HUVECs for 8 h with plasma‐sEVs of HC and PT‐patients (isolated 4 and 24 h after trauma). N‐numbers indicate independent samples. Statistical tests: a) Ordinary one‐way ANOVA with Dunnett's multiple comparison test; f) Two‐tailed unpaired Student's *t*‐test. *P < 0.05; **P < 0.01; ***P < 0.001, ****P < 0.0001; ns: no significant difference.

In order to demonstrate the relevance of our findings for patients, we have analyzed polytrauma patient (PT)‐plasma (injury severity score,^[^ ^58^ ^]^ ISS ≥ 16; Table S1, Supporting Information). Plasma samples from six patients were acquired at the time of shock room admittance (T0) and after 4, 12 as well as 24 h. Subsequently, sEV concentrations were determined from equal volumes and normalized to total protein levels, while plasma of healthy individuals with comparable age served as control (HC). Interestingly, sEV concentrations were significantly increased 24 h after polytrauma (**Figure** **9a**). To evaluate molecular functions, RNAseq was performed on HUVECs treated for 8 h with respective sEVs isolated 4 and 24 h after polytrauma in respect to HC (Supporting Information Data File 10–12). Although data suffered from noise and variances, likely caused by different injury patterns or ER‐admittance times, we were able to identify 113 significantly regulated transcripts upon treatment with the 4 h‐sEVs. EnrichR meta‐analysis with low threshold levels (log_2_ ≥ 0.135 log_2_ ≤ ‐0.135) allowed for the inclusion of weakly regulated transcripts and indicated 41 up‐ and 44 downregulated genes. For the 24 h time‐point, 231 transcripts were significantly changed: 89 up‐ and 114 downregulated. PCA showed clearly separated clusters of HC, whereas samples treated with PT‐sEVs were overlapping (Figure 9b). Unsupervised clustering demonstrated matching gene regulation for 4‐ and 24 h PT‐samples (Figure 9c). In line, EnrichR meta‐analysis of the 4 h‐sEV treatment identified terms associated with plasminogen activation, vascular inflammation, and RAGE‐signaling,^[^ ^59^ ^]^ CXC‐chemokine signaling, NF‐*κ*B, positive regulation of leukocyte chemotaxis, and ARDS, whereas terms associated with cholesterol biosynthesis were depleted (Figure 9d; Supporting Information Data File 10). At the 24 h time‐point, terms associated with plasmin signaling, blood coagulation, NF‐kB, PDGF, TGF‐*β*, the Fra‐pathway implicated in pulmonary fibrosis^[^ ^60^ ^]^ as well as chemokine/inflammatory signaling, that is, S100A8‐complex^[^ ^61^ ^]^ or regulation of IL1*β*‐expression targets^[^ ^62^ ^]^ were identified (Figure 9e; Supporting Information Data File 10). We therefore investigated whether transcripts previously identified in vitro and in TxT mice would also be enhanced in PT‐sEV‐treated HUVECs. Intriguingly, transcripts for ICAM‐1, VCAM‐1, SELE, CXCL2, and CXCL8 were significantly increased at both time points (Figure 9f). Thus, we show the same targets are addressed by sEVs of the *in‐vitro* trauma system, TxT mice, and in polytrauma patients. In conclusion, our data demonstrate an unexpected function of sEVs in the propagation of inflammation after physical trauma, involving the endothelium and neutrophils. Interestingly, inhibition of sEV‐release after TxT using the inhibitor GW4869 in mice improved multiple readouts for trauma outcome.

## 3 Discussion

Traumatic injury is the most common cause of death in younger individuals. There are various traumatic injuries, including physical insults and hemorrhage but also acute bacterial and viral infections. A common feature of all these traumatic insults is the occurrence of an excessive, imbalanced immune response. Dysregulated inflammation is mainly driven by high levels of cytokines, chemokines, and anaphylatoxins, resulting in complications such as systemic inflammatory response syndrome (SIRS) or MODS.^[^ ^2^, ^63^ ^]^ However, the mechanisms that cause a normal immune response to evolve into exacerbated, detrimental inflammation are incompletely understood and efficient treatment options are rare. To address this issue, we were prompted to unravel alternative routes of immune activation by investigating sEVs and their potential functions in driving the inflammatory traumatic response. We decided to investigate sEVs, since previous studies have shown their immunomodulatory capacity.^[^ ^52^, ^64^ ^]^ However, a potential role of sEVs in mediating the response to severe injury has so far not been studied in detail. Here, we demonstrate sEVs are crucial mediators of local and systemic posttraumatic inflammation. Our data indicate that TxT or HS significantly increased sEV secretion (Figure 1a‐f) and we identify endothelial cells as well as neutrophils as a major source of trauma‐induced sEV release (Figure 1g). By investigating TxT and HS, which are both major activators of systemic inflammation,^[^ ^65^ ^]^ we specifically aimed at evaluating different, severe traumatic insults to show that increased sEV‐release is a generalized response. The in vivo data are supported by experiments using an in vitro trauma model where a posttraumatic microenvironment is induced by treatment with a polytrauma cocktail (PTC), comprising cytokines and anaphylatoxins at concentrations detectable in polytrauma patients.^[^ ^22^ ^]^ Utilizing PTC, we demonstrate that increased sEV secretion in HUVEC cells is caused by a concomitant increase in ESCRT‐dependent and ‐independent Rab‐GTPases,^[^ ^27^ ^]^ which also control sEV release in the TxT mouse model (Figures 1h,i and 2b‐e). Here, IL1*β* is likely the main driver (Figure 2d), in line with a finding by Willis et al. showing enhanced sEV secretion from astrocytes upon IL1ß treatment.^[^ ^66^ ^]^ Importantly, PTC‐sEVs alone were able to trigger inflammatory phenotypes in HUVEC cells and facilitated increased adhesion of PMNs to the endothelium (Figure 3a,b). Mechanistically, we show that a direct transfer of mRNAs encoding for CAMs and cytokines into recipient cells is responsible for the propagation of inflammation. Functional mRNA transfer from sEVs has already been reported previously,^[^ ^67^ ^]^ however, it was an open question whether the sEVs investigated in this study contained only fragments or indeed functional full‐length transcripts. Thus, we abolished the potential delivery of intact mRNA by RNAse treatment of sEVs (Figure 3c), performed in vitro translation experiments (Figure S1k, Supporting Information), and examined protein expression upon sEV‐transfer by Western blot or ELISA (Figure 3e,f). Using MS and RIP‐experiments, we demonstrate that the sEV‐resident mRNA binding protein WDR1^[^ ^31^ ^]^ was implicated in the loading of CAM and cytokine transcripts into sEVs upon PTC‐treatment of HUVECs (Figure 4a‐e). While CAM and cytokine mRNAs are enriched in WDR1‐RIPs, the abundant non‐inflammatory transcript GAPDH was comparatively depleted in RIPs of PTC‐treated samples (Figure 4e). Enrichment of inflammatory transcripts in PTC‐treatment WDR1‐RIP assays thus suggests a replacement of non‐inflammatory transcripts, such as GAPDH with inflammation‐associated targets. Moreover, we identified additional RBPs that were also increased and may have supporting functions (Figure 4b,c). Besides, our data show that PTC‐sEVs not only educate endothelial cells, but also primary PMNs by enhancing the expression of specific targets including CXCR2, PECAM‐1 CEACAM8, and SELP. This leads to a further amplification of endothelium‐neutrophil interactions that are known to facilitate migration and extravasation, thereby promoting the inflammatory cascade (Figure 4f).^[^ ^32a‐c^ ^]^ PTC‐sEVs also disrupt the endothelial barrier by a mechanism primarily dependent on destabilization of VE‐cadherin‐mediated cell‐cell contacts^[^ ^34^ ^]^ via downregulation of CTNND1 (p120‐catenin) through mir‐298 (Figure 4g,h and 5a‐i).^[^ ^23^, ^36^ ^]^ A plethora of miRNAs are discussed as potential regulators of endothelial permeability.^[^ ^68^ ^]^ Here, we describe a novel function of mir‐298 in barrier destabilization by in vitro and in vivo “trauma‐sEVs.” We also detected high levels of MVP,^[^ ^39a^ ^]^ a dedicated miRNA binding protein^[^ ^31^ ^]^ in PTC‐sEVs (Figure 4b; Figure S2c, Supporting Information). This suggests that MVP may be involved in the loading of endothelial barrier‐relevant miRNAs, a hypothesis that shall be examined in detail in further studies.

These findings pointed to a general role of sEVs in mediating and amplifying inflammation after traumatic injury. Therefore, we examined whether blocking sEV‐release in vivo might be a potential novel therapeutic approach to prevent trauma‐induced injuries in a clinically relevant setting, that is, 10–15 min after the occurrence of the traumatic insult. The sEV‐biogenesis inhibitor GW4869^[^ ^40^, ^52^ ^]^ was used in vivo in mice in a time frame that corresponds to an early medical intervention after trauma in a real‐world setting, decreasing sEV concentrations 4 h after TxT (Figure 6b). This drastically reversed enrichment of trauma‐associated signatures on a whole transcriptome and proteome level in TxT lungs (Figure 6c‐f; Figures S2d‐f and S3a‐b, Supporting Information). In particular, the expression of cytokines and CAMs, previously identified in vitro, was reverted upon GW4869 injection, concomitant with a significantly impaired PMN infiltration (Figure 6h‐j). We also found that signs of systemic inflammation, endothelial glycocalyx breakdown, barrier destabilization as well as evidence of distant organ damage in kidneys (AKI) were reversed by GW4869 (Figure 7a‐c). Targets in mice were further corroborated in vitro by treatment of mouse primary lung microvascular endothelial cells with TxT‐sEVs (Figure 7d). Moreover, we detected a significant downregulation of Ctnnd1 as well as increased mmu‐mir‐298‐5p/‐3p^[^ ^36^ ^]^ (Figure 7e‐g) expression in samples treated with TxT‐sEVs and effects of the respective sEVs on Ctnnd1 expression in primary lung microvascular endothelial cells could be abrogated using a mmu‐mir‐298‐5p inhibitor (Figure 7f).

GW4869 is a neutral sphingomyelinase (nSMase) inhibitor and the most widely used pharmacological agent for blocking exosome generation.^[^ ^52^, ^64^, ^69^ ^]^ GW4869 inhibits the ceramide‐mediated biogenesis of intraluminal vesicles in MVBs and the release of mature exosomes from MVBs.^[^ ^53^ ^]^ Bioactive sphingolipids, such as ceramide, are also important signaling molecules in the autonomous nervous system and thus are able to contribute to a balanced inflammatory response.^[^ ^70^ ^]^ Ceramide is produced through various pathways, but the hydrolysis of sphingomyelin by sphingomyelinases (SMases) plays a predominant role. nSMase2 is a crucial enzyme involved in multiple cellular pathways, for example, cell cycle arrest and exosome biogenesis.^[^ ^71^ ^]^ nSMase2 is also involved in the TNF‐*α* mediated secretion of inflammatory markers, like IL‐1*β* and MCP‐1 from macrophages and monocytes.^[^ ^72^ ^]^ However, in our endothelial in vitro trauma system, TNF‐*α* is not part of the PTC cocktail and thus molecular targets identified in vitro upon transfer by sEVs that were also verified in vivo after treatment of TxT mice with GW4869, are more likely regulated by inhibiting sEV‐release. Additionally, we have controlled the effects of GW4869 treatment along with PTC‐stimulation on HUVEC cells and did not see any direct downregulation, but rather increased levels for the pro‐inflammatory transcripts, such as ICAM‐1, VCAM‐1, and E‐Selectin (Figure S4c, Supporting Information). This is likely caused by the accumulation of the respective mRNA in cells, due to the blocking of sEV release. Furthermore, we have validated the role of sEVs as inflammatory mediators in vivo by analyzing molecular cargo transfer to primary endothelial cells (Figure 7d‐h) and upon reinjection of purified sEVs in mice (Figure 8). A role of sEVs in the regulation of inflammation utilizing GW4869 so‐far has only been studied as part of specialized inflammatory disorders, such as neuroinflammation and lipopolysaccharide (LPS)‐induced cardiac dysfunction.^[^ ^40^, ^52^, ^73^ ^]^ To our knowledge, GW4869 has not been used in a trauma setting to manage a systemic exaggerated immune response caused by a severe injury that is propagated by endothelial‐derived sEVs. Also, no clinical studies have been described so far with the GW4869 small molecule inhibitor.

An alternative way to block sEV‐biogenesis in experiments and support inhibitor data would have been knockout of Rab27a in vitro and in vivo.^[^ ^74^ ^]^ However, since sEV biogenesis in our system was controlled by Rab27a‐ and Rab11b, ablation of only Rab27a would not have sufficed. We also did not opt for a genetic disruption of Rab11b, due to broad effects on endosomal recycling^[^ ^75^ ^]^ that would have obscured any meaningful molecular or phenotypic analysis. Moreover, we purposely opted to utilize the GW4869 inhibitor for in vivo experiments in order to address the clinical relevance of our study design^[^ ^76^ ^]^ by preventing an acute hyperinflammatory response after TxT. Yet, we acknowledge that further studies will be necessary to investigate questions, such as optimal therapeutic windows and long‐term effects of bolus inhibitor injections after severe thoracic injury. In order to further corroborate a crucial role of sEVs in propagating systemic inflammation, independently of inhibitor treatment, we have also administered purified plasma‐sEVs from TxT mice into healthy animals (Figure 8), which in turn induced inflammation, regulation of barrier‐relevant proteins in lungs, increased infiltration of PMNs, and facilitated systemic cytokine release, as well as signs of AKI (Figure 8). Although increased sEV release was facilitated by both ESCRT‐dependent and ‐independent pathways in our study, GW4869,^[^ ^40^, ^52^ ^]^ which preferentially targets ESCRT‐independent ceramide‐mediated sEV‐biogenesis, was able to revert adverse trauma‐associated phenotypes and molecular signatures in vivo. Given our results, it would thus be tempting to block the peak sEV‐release after severe TxT and/or remove posttraumatic sEVs from patient plasma using extracorporeal hemofiltration devices, as suggested in previous studies for other inflammatory conditions.^[^ ^77^ ^]^ Besides the inhibitor and sEV injection experiments in TxT mice, the clinical relevance of our findings was demonstrated by significantly increased plasma‐sEV concentrations PT patients (PT) 24 hrs after ER admittance compared to HC ( Figure 9a). This is in line with a descriptive study from Kuravi et al., where the authors reported significantly enhanced levels of EVs from endothelial cells and leukocytes in the plasma of trauma patients.^[^ ^14b^ ^]^ Upon investigating the role of PT‐patient sEVs in endothelial reprogramming in vitro, as interrogated by our RNAseq studies, we also identified enrichment of signatures associated with inflammation, endothelial activation, and leucocyte transmigration (Figure 9d). These data were corroborated by verifying the expression of CAM and cytokine transcripts, previously observed in vitro and in mice (Figure 9b‐f).

## 4 Conclusions

**Figure 10:**
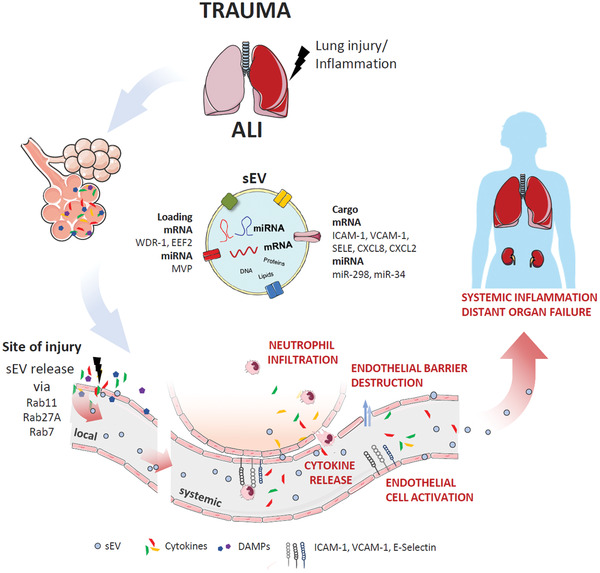
Scheme for the role of endothelial sEVs in post‐traumatic inflammation.

In conclusion, our data indicate a prominent, previously unrecognized role of sEVs in the transmission and amplification of acute inflammation after severe thorax trauma, even contributing to distant organ failure, but also imply a more universal role of sEVs in mediating inflammation after different traumatic insults. Blocking sEV release to abrogate an acute posttraumatic hyperinflammatory response in vivo may therefore constitute an interesting option to normalize inflammation and improve trauma outcome (**Figure** **10**).

## 5 Experimental Section

### Key Materials

Key materials are listed in Table S2, Supporting Information.

### Ethics Committee Approval

Local ethics committee approval: 116/14 for peripheral blood from healthy probands, 94/14 for peripheral blood from polytrauma patients. Patient and proband data are listed in Table S1, Supporting Information.

### Thorax Trauma (TxT) Mouse Experiments

All mouse experiments were performed in adherence with the National Institute of Health Guidelines on the Use of Laboratory Animals and the European Union “Directive 2010/63/EU on the protection of animals used for scientific purposes” (animal experiment approval number: 1413, 1258).

### Animal Preparation

Twelve‐week‐old, male C57BL6 mice were anesthetized with sevoflurane (3.5%, Abott, Wiesbaden, Germany) and oxygen (96.5%) mixture under a continuous flow of 0.8 L min^–1^. Buprenorphine (Essex, Pharma, Munich, Germany) was administered subcutaneously (0.05 mg kg^–1^ body weight) 30 min prior to the trauma or sham procedure. Animals were allowed free access to water and food before and after experimental procedures. The animals were observed frequently during the experiment. During the observation period of 4 h, the animals were evaluated on the basis of the following criteria for euthanasia: hypothermia, cyanosis, shivering, seizures, loss of motion, and paralysis. At the end of the observation period, the anesthetized mice (3.5% sevoflurane and 96.5% oxygen mixture under a continuous flow of 0.8 L min^–1^) were euthanized by exsanguination via cardiac puncture. *Blunt Chest Trauma*: Bilateral lung contusion was induced by a single blast injury in anesthetized mice as described previously.^[^ ^9^, ^78^ ^]^ In brief, the opening of a high‐speed valve (Hee‐D‐24, Festo, Esslingen, Germany) delivered compressed air into the upper section of a cylinder. The upper compartment is separated to the lower section with a Mylar polyester film (Du Pont de Nemur, Bad Homburg, Germany). The polyester membrane ruptured at standardized pressure releasing a defined blast wave in the lower compartment of the cylinder, centered on the ventral thorax of the animal. The level of pulmonary contusion was chosen based on histologic, cardiopulmonary as well as immunologic changes in earlier studies and was sufficient to induce a profound local and systemic inflammatory response, but without being lethal itself.^[^ ^9^ ^]^ Ten to 15 min after the blast injury GW4869 (Sigma Aldrich #D1692, 2 µg g^–1^ body weight in 0.9% NaCl) or NaCl was administered via tail‐vein injection.^[^ ^40^ ^]^

### Hemorrhagic shock (HS) Experiments in FBM Pigs

The HS experiments in pigs were approved by the University of Ulm Animal Care Committee and the Federal Authorities for Animal Research. The experiments were performed in adherence with the National Institute of Health Guidelines on the Use of Laboratory Animals and the European Union “Directive 2010/63/EU on the protection of animals used for scientific purposes” (animal experiment approval number: 1341). For the experiments, blood from four familial hypercholesterolemia Bretoncelles Meishan (FBM) pigs was used from a greater study of 23 animals. The FBM pig strain is a cross‐bread of Rapacz pigs with smaller strains (Chinese Meishan and Bretoncelles), characterized by a homozygous R84C low‐density lipoprotein (LDL) receptor mutation. Under high‐fat diet (1.5% cholesterol, 20% bacon fat), the animals develop substantial hypercholesterolemia and subsequent atherosclerosis with coronary artery disease (CAD). Animals received the diet (20 g kg^‐1^ d^‐1^) for at least 9 months prior to the experiments. Animals had a median age of 21 months (interquartile range (IQR): 18 to 23 months) and a median weight of 77 kg (IQR: 72–83 kg). Anesthesia and surgical instrumentation of the swine as well as the volume‐ and pressure‐controlled HS protocol were performed as recently described.^[^ ^79^ ^]^ Blood samples were taken immediately after induction of anesthesia (“pre‐shock and surgical trauma”) and after resuscitation from the 3 h HS (“post‐shock”) at 17, 41, and 65 h.

### Cell Culture and Primary Cells

Human umbilical vein endothelial cells (HUVEC, ATCC, CRL‐1730) were maintained at 37°C and 5% CO_2_ in endothelial cell growth medium MV2 (PromoCell, #C‐22022). C57BL/6 mouse primary lung microvascular endothelial cells were purchased from Pelo Biotech (#PB‐C57‐6011) and maintained as described above in complete mouse endothelial cell medium (Cell Biologics, #M1168).

### Plasma Preparation from Mice

Blood obtained by cardiac puncture from mice was placed in EDTA tubes (plasma) or untreated Eppendorf tubes (serum) and spun at 1500 rpm for 10 min. Plasma/serum was transferred in Lo‐bind tubes (Eppendorf, #00 301 08116) and stored at ‐80 °C.

### Preparation of Tissue Lysates

Tissue specimens were cut in small pieces, shock frozen on dry ice, and stored at ‐80 °C. For the preparation of protein lysates pieces of tissue with a weight of ≈20 mg were transferred in 2 mL reaction tubes. Lysis buffer (150 × 10^–3^ m sodium chloride, 1% NP‐40, 50 × 10^–3^ m Tris pH 8.0, 1× Protease Inhibitor Cocktail [Sigma–Aldrich, #11 873 580 001], 1× Phosphatase Inhibitor Cocktail [Sigma–Aldrich, #4 906 837 001]) was added in relation to the amount of tissue (24 µL per 1 mg of tissue). Sterile metal beads were added to the tubes and tissues were ground with an electric homogenizer (Qiagen) at a frequency of 50 s^–1^ for 5 min. Samples were centrifuged for 20 min at 12 000 rpm at 4 °C. Supernatant was transferred to a new tube and stored at ‐80 °C for further analysis. Equal amounts of protein extracts were fractionated by SDS‐PAGE, transferred to nitrocellulose or PVDF membranes, and sequentially probed with the appropriate primary antibodies (see essential resource list). Protein was detected using SuperSignal West Dura reagent (ThermoFisher Scientific, #34 076). Act in was used as a protein loading control to ensure equal loading.

### RNA Isolation from Tissue

Small pieces of tissue specimens were transferred in tubes containing 700 µL QIAzol lysis reagent. Sterile metal beads were added to the tubes and tissues were ground with an electric homogenizer (Qiagen) at a frequency of 50 s^–1^ for 5 min. Samples were centrifuged for 20 min at 12 000 rpm at 4 °C. The supernatant was transferred to a new tube and RNA was isolated with the miRNeasy® Mini Kit (Qiagen, #217 004) according to the manufacturer's instructions.

### sEV Isolation from Serum/Plasma

Blood samples were incubated at RT for 10 min. Subsequently, serum or plasma was separated by centrifugation at 2000 rpm for 10 min at RT, followed by the centrifugation at 13 000 rpm for 20 min. Serum/plasma was transferred into a new LoBind 1.5 mL tube on ice and then subjected to SEC using Exo‐Spin^TM^ columns (Cell Guidance Systems) according to the manufacturer's description. In brief, Exo‐spin^TM^ columns (Cell Guidance Systems, #EX03‐50) were centrifuged for 30 s at 500 rpm after cap and stopper were removed. The columns were equilibrated by centrifugation at 500 rpm for 30 s using sterile filtered PBS (200 µL). One hundred microliters of serum/plasma was directly loaded onto a column and centrifuged for 1 min at 500 rpm. The eluate was discarded, and the column was placed in a fresh 1.5 mL‐LoBind tube. For elution, sterile‐filtered PBS (200 µL) was added to the column and centrifuged for 1 min at 500 rpm. The sEV eluate was stored at ‐80 °C.

### Bronchoalveolar Lavage Fluid (BAL)

At the end of the study 4 h after blunt chest trauma, animals were sacrificed, the trachea of the animals was carefully exposed and cannulated using 0.58 mm × 0.2 mm internal diameter polyethylene tubing (Merck, Darmstadt, Germany). Then, (0.7 mL) ice‐cold PBS was carefully injected and recovered three times and stored on ice after the addition of 1 µL of proteinase inhibitor cocktail per 100 µL of BAL. BAL was centrifuged at 500 × *g* for 10 min at 4 °C. The supernatant fluid was stored at ‐80 °C until analysis. The pellet was resuspended in PBS (200 µL). Fifty to 80 µL of cell suspension (dependent on pellet size) was centrifuged on cytospin slides at 600 rpm for 3 min. For fixation one drop of 100% methanol was added directly to the cells.

### Differential Staining of BAL Cytospin Slides

For analysis of lung resident cells in BAL, cytospin slides were differentially stained according to Pappenheim's protocol. Cells were first covered for 10 min with methanol before May‐Grünwald solution (VWR, 35 262), 1:2 diluted in phosphate buffer, was added for 7 min. Subsequently, slides were stained with Giemsa solution (VWR, 35 260) and finally washed three times in phosphate buffer (pH 6.8, 10 min, 2 min, 2 min). Samples were analyzed by brightfield microscopy (Keyence, BZ‐9000, PlanApo_VC60xH 1.40/0.13 mm Oil) with ten images per slide. Cells were counted manually according to specific morphology to determine the number of infiltrated immune cells in the BAL.

### sEV Isolation from Cell Culture Supernatants

sEVs were purified by precipitation and size SEC using the Exo‐Spin Kit (Cell Guidance Systems, EX01‐50). A total of 1 × 10^6^ cells were seeded in 15 cm^2^ dishes. After cells reached confluency, cells were washed several times with serum‐free DMEM and then incubated in serum‐free media for 24 h to accumulate sEVs at 37 °C, 5% CO_2_. Culture mediums were collected and centrifuged for 15 min at 1000 rpm at 4 °C. Subsequently, the culture supernatants were centrifuged for 45 min at 4000 rpm (4 °C) and then passed through 0.2 µm syringe filters (Filtropur S, #83.1826.001, Sarstedt). The supernatant was concentrated by ultrafiltration at 2000 rpm and 4 °C using Vivaspin Turbo 15 (100 000 MWCO) units (Sartorius, #VS15T41) until a volume of 1 mL was reached. The concentrated supernatant was transferred into LoBind 1.5 mL tubes and 0.5 volumes (V) of supplied buffer A was added. After incubation overnight at 4 °C, the samples were centrifuged for 1 h at 14 000 rpm and 4 °C. The supernatant was discarded, and the pellet was resolved in sterile (0.2 µm) filtered PBS (100 µL). For SEC, Exo‐spin columns were centrifuged for 30 s at 500 rpm after cap and stopper were removed. The columns were equilibrated by centrifugation at 500 rpm for 30 s using sterile filtered PBS (200 µL). The sEV pellet solved in PBS was pipetted onto the column and centrifuged for 1 min at 500 rpm. The eluate was discarded, and the column was placed in a fresh 1.5 mL‐LoBind tube. For elution, sterile‐filtered PBS (200 µL) was added to the column and centrifuged for 1 min at 500 rpm. The sEV eluate was stored at ‐80 °C. The surface protein concentration was determined using Bradford Protein Assay (Bio‐Rad, #5 000 006). For purity analysis of sEV‐samples isolated by the Exo‐spin Kit, solubilized sEVs were pelleted by 100 000 × *g* ultracentrifugation for 2 h at 4 °C (Beckman Coulter Optima‐Max‐XP, rotor MLA‐130). Supernatant and dissolved pellet fractions were subsequently used to treat HUVEC cells for 8 h.

### TCL After sEV Secretion

TCL of the remaining cells after sEV secretion were prepared by washing the cells followed by lysis using 1 mL lysis buffer‐1 (50 × 10^–3^ m Tris, pH 7.4, 150 × 10^–3^ m NaCl, 5 × 10^–3^ m MgCl_2_, 1% Triton X‐100, supplemented with 1× Protease Inhibitor Cocktail and 1× Phosphatase Inhibitor Cocktail. Lysates were incubated for 20 min on ice and cleared by centrifugation (20 min, 13 000 rpm at 4 °C). The supernatant was transferred to a new tube and stored at ‐80 °C. Protein concentration was determined using the Bradford Protein Assay. For protein analysis, samples were subjected to SDS‐PAGE on acrylamide gels (10%) and transferred onto nitrocellulose membranes. Blots were blocked with BSA (2%) in TBS‐Tween‐20. Primary antibodies were applied in blocking solution overnight at 4 °C. The membranes were incubated with the appropriate secondary antibodies conjugated to horseradish peroxidase (HRP) for 1–2 h at room temperature, signal was detected using SuperSignal West Dura reagent (ThermoFisher Scientific, #34 076).

### Bradford Protein Assay with sEVs

The Bradford reagent (Bio‐Rad) was diluted 1:5 in water. To diluted Bradford reagent (1 mL), 1–10 µL sEV solution in PBS or total cell lysate was added into a cuvette and measured using a photometer (OD595). The concentration was determined by the OD595 value using a calibration curve with BSA protein.

### Nanoparticle‐Tracking Analysis (NTA) of sEVs

NTA was performed using Nanosight LM10 or NS300 devices as well as the respective software (Malvern Panalytical). Isolated sEVs were diluted in particle‐free sterile filtrated PBS, held in a syringe, and injected into the Nanosight tracking chamber. Capturing options were set to 60 s at a camera gain of 560 (LM10). For measurements with the NS300 device, samples were injected using a syringe pump and particles were tracked under constant flow. Each sEV sample was measured three times for 60 s. For background measurements, sterile filtrated PBS was injected into the chamber and measured using the same conditions.

### sEV Uptake Assays

sEV uptake experiments were performed by labeling 20 µg of purified sEV using the ExoGlow‐Membrane™ EV Labeling Kit (Cat #EXOGM600A‐1, SBI Systems Bioscience). sEV uptake was measured by incubating the indicated amount of labeled sEVs with 1 × 10^5^ cells in serum‐free media for 4 h in a cell culture incubator. Subsequently, cells were washed and live cells were analyzed by flow cytometry in the PE‐CY5‐A channel for the extent of Exo‐Glow labeling.

### Isolation of RNA from sEVs

For purification of total RNA including miRNA 700 µL QIAzol was added to sEVs derived from cell culture supernatants or serum/plasma. Subsequently, isolation was performed using the miRNeasy Micro Kit (Qiagen, #217 084)

### Transmission Electron Microscopy (TEM) of sEVs

sEVs were prepared for TEM by negative staining. Therefore, 5 µL of the exosome solution was added onto a glow discharged carbon‐coated copper TEM grid and incubated for 1 min. Then the grids were washed three times with a droplet of water. Grids were incubated three times with droplets of 3% uranyl acetate in water for 30 s each. Afterward, grids were dried on air with a small amount of uranyl acetate left. The grids were imaged with a JEOL 1400 TEM.

### Quantitative Real‐Time PCR

RNA was extracted from homogenized tissue or cells with the miRNeasy® Mini Kit (Qiagen, #217 004) according to the manufacturer's instructions. cDNAs were prepared from 250 ng of total RNA, using the iScript™ cDNA Synthesis Kit (Biorad, #1 708 891) for mRNA or the miScript II RT Kit (Qiagen, #218 161) for miRNA. qPCR reactions were performed with PowerUp^TM^ SYBR^TM^ Green Master Mix (Applied Biosystems, #A25742) to analyze mRNA expression or miSript SYBR Green PCR kit (Qiagen, #218 073) for the abundance of miRNA according to the manufacturer's instructions. The thermal cycling conditions were composed of 2 min at 95 °C, followed by 45 cycles of 15 s at 95 °C denaturation and 1 min at 60 °C anneal/extension. The relative gene expression levels were calculated by the “delta‐delta Ct‐method” (ΔΔCt method).

### RNA‐Binding Protein Immunoprecipitation Assay

RNA‐binding protein immunoprecipitation assay (RIP) assays were performed using the EZ‐Magna RIP RBP Immunoprecipitation (IP) kit (Merck, 17–701) according to the manufacturer's description. To precipitate the RBP WDR1, three confluent 15 cm dishes of HUVEC cells were either treated with vehicle or PTC for 24 h and subsequently used to generate total cell lysates for a single IP reaction. For immunoprecipitation, either 10 µg of WDR1 antibody or normal non‐specific mouse IgG (provided in the kit) was used as a negative control. Co‐precipitated RNA was isolated using the miRNeasy Micro Kit (Qiagen, #217 084), and qPCR was performed as described above.

### Isolation of Human PMNs

Venous blood from healthy donors was collected in EDTA monovettes. Five milliliters of blood was overlayed on 5 mL of Polymorphrep (Axis Shield, #AXS‐1114683) in a 15 mL tube and centrifuged at 500 × *g* for 30 min at RT. The band containing PMNs was transferred into a new tube and mixed with the equal volume of half‐strength Hepes‐buffered saline (0.85%, w/v, NaCl, 20 × 10^–3^ m Hepes‐NaOH, pH 7.4). PMNs were pelleted (400 x *g*, 10 min) and resuspended in 5 mL Hepes‐buffered saline. To count the PMNs 50 µL of cell suspension was mixed with Turcks solution (500 µL). After 5 min cells were counted in a Neubauer chamber.

### PMN Adhesion Assay

HUVECs were seeded in black clear‐bottom 96‐well plates (20.000/well) and grown for 2 days until the endothelial monolayer was confluent. PMNs were isolated from fresh human blood and resuspended in serum‐free medium. Cells were stained with CellTracker Deep Red (2 × 10^–6^ m, Thermo Fisher scientific, #C34565) for 30 min at 37 °C. Cells were spun down for 10 min at 400 × *g* and resuspended in serum‐free medium. Fluorescence‐labeled PMNs were then added to the HUVEC monolayers and incubated for 20 min before the plate was washed five times with PBS. Adhesion of remaining PMNs was analyzed by scanning fluorescence measurements (extinction/emission 630/660) using a Tecan M200Pro plate reader.

### Transendothelial Electrical Resistance (TEER)

TEER was measured by impedance spectroscopy using the cellZscope (NanoAnalytics, Münster, Germany). A total of 100 000 HUVEC cells were seeded on Corning Costar Transwell cell culture inserts (5 µm pore size, Costar #3421) and grown for 2 days. For measurements, the basal electrode was overlaid by 500 µL equilibrated medium. After inserting filters, 275 µL of medium were added to the apical surface and the apical electrode was placed into the apical liquid. The measurements were performed immediately after positioning of apical electrodes. The software package provided with the instrument (NanoAnalytics, Münster, Germany) was used for data acquisition and analysis.

### Transendothelial Flux Assay with FITC‐Albumin

Endothelial barrier stability was evaluated by measuring the flow of fluorescein isothiocyanate‐conjugated albumin (FITC‐Albumin, Sigma–Aldrich, #A9771) across confluent, differentiated HUVEC monolayers. For FITC‐albumin flux assays, 100 000 HUVECs were seeded on Corning Costar Transwell cell culture inserts (5 µm pore size, Costar #3421). After 72 h, with daily medium exchange, the medium in the bottom wells was replaced with phenol red‐free endothelial cell growth medium. Subsequently, 100 µL phenol red‐free medium supplemented with 40 µg mL^–1^ FITC‐Albumin was added to the filter inserts. At indicated time points, 50 µL of the medium was removed from the basal compartments and transferred into black 96‐well plates. FITC fluorescence (excitation/emission 490/520) was measured using a Tecan M200Pro plate reader.

### Immunofluorescence and Quantitative Analysis

For confocal imaging, cells were processed as described previously.^[^ ^80^ ^]^ In brief, 1.5 × 10^5^ HUVEC cells were seeded on sterilized 15 × 15mm coverslips in 12‐well plates and grown to confluency. Subsequently, HUVEC monolayers were treated with 2 µg of sEVs for 8 h. For miRNA transfection experiments, HUVECs were transfected with 25 × 10^–9 ^mol miR‐mimic or respective controls using Amaxa HUVEC Nucleofector Kit (Lonza, # VVPB‐1002). A total of 1×10^5^ cells were seeded in 96‐well imaging plates. Samples were fixed using formaldehyde (3.75%) for 20 min at RT and permeabilized with Triton X‐100 (0.1%) in PBS for 30 s. After blocking (5% FCS, 0.05% Tween‐20 in PBS) for 20 min, samples were stained with primary antibody for 2 h at RT or overnight at 4°C. Samples were washed three times with PBS and secondary antibodies conjugated with an Alexa fluorophore (Thermo Scientific) were applied for 1 h at room temperature. F‐actin was stained using Phalloidin‐Alexa‐Fluor‐647 (1:60; Thermo Scientific). Nuclei were stained with 4′,6‐Diamidin‐2‐phenylindol (DAPI). Coverslips were mounted in Fluoromount‐G (Thermo Scientific, #00‐4958‐02). Quantitative intensity quantification was performed as described.^[^ ^81^ ^]^ Experiments were analyzed by a confocal laser scanning microscope TCS‐SP8‐HCS (Leica, Mannheim, Germany) equipped with an HC PL APO CS2 40×/1.3 oil immersion objective. Images were acquired in sequential scan mode. Gain and offset were set in a way that all cellular structures were imaged within the linear range of detectors having no saturated pixels and black level background values using HyD‐detectors. Quantitative mean of ROI analysis was conducted utilizing NIH ImageJ. The same conditions were applied to all images.

### Multiplex Cytokine/Chemokine Analysis

Bead‐based multiplex cytokine analysis was done according to the manufacturer's instructions using either 36‐plex cytokine/chemokine procartaplex panels (ThermoFisher Scientific, EPX360‐26092‐901) or custom mouse 10‐plex panels (IL1*β*, TNF‐*α*, GRO*α*, ENA78, MCP1, MCP3, MIP1*α*, MIP1*β*, MIP2*α*; ThermoFisher Scientific) and the Luminex 200 device. Outliers were removed based on a ROUT outlier test (Q = 5%).

### Enzyme‐Linked Immunosorbent Assays

Enzyme‐linked immunosorbent assays (ELISA) were performed according to the manufacturer's instructions. For human IL6 (ThermoFisher Scientific, #BMS213‐2) and CXCL8 (ThermoFisher Scientific, #KHC0081) total cell lysates from HUVECs were used. Mouse IL1*β* (R&D Systems, #MHSL1300), CD138 (Diaclone, #860.090.096), and NGAL (R&D Systems, #MLCN20) ELISAs were performed with plasma from TxT mice in tested concentrations. Standard curves were created using nonlinear 4P regression.

### RNaseA Treatment of sEVs

sEVs were isolated from cell culture supernatants after 24 h of vehicle or PTC treatment. For permeabilization, saponin was added to sEV samples to achieve a final concentration of 0.5%, the equal amount of H_2_O was used as a negative control. Subsequently, RNAse A (ThermoFisher, #AM2269) with a final concentration of 20 µg mL^–1^ was added and samples were incubated at 37 °C for 10 min. To stop the reaction, RNase inhibitor (1 U mL^–1^ Thermo Fisher, #N8080119) was used and sEV samples were incubated at 55 °C for 5 min. Corresponding sEV samples were immediately subjected to HUVECs for 8 h before RNA was isolated from these cells for qPCR analysis.

### Knockdown Experiments

For nucleofection of siRNAs in HUVECs, the human umbilical vein nucleofector kit (Lonza # VVPB‐1002) was used according to the manufacturer's instructions. Briefly, 500 000 cells were pelleted and resuspended in 100 µL nucleofector solution supplemented with 100 pmol siRNA before the mixture was transferred into the cuvette. Then, the cuvette was inserted into the nucleofector and the program U‐001 was applied. Immediately after the program finished, a pre‐equilibrated medium (500 µL) was added and the sample was transferred into a 15 cm dish with 15 mL medium. For one 15 cm dish, the procedure was repeated twice. Cells were incubated for 30 h before the medium was exchanged with serum‐free medium and sEVs were isolated after additional 24 h as described.

### In Vitro Translation

RNA was isolated from vehicle and PTC‐sEVs as described. The total amount of RNA was used for *in‐vitro* translation with the rabbit reticulocyte lysate system (Promega, #L4960) and non‐labeled complete amino acid mixture (Promega, #L4461) according to the manufacturer's description. After the translation reaction, samples were supplemented with lämmli buffer and incubated for 5 min at 95°C to enable protein analysis via WB.

### G3 Human miRNA Microarray Analysis of sEVs—Isolatioon of Total RNA Including miRNAs from sEVs

For microarray analyses, we isolated total RNA, including miRNA from sEVs using the miRNeasy® Micro Kit, (Qiagen, # 217 084) according to the manufacturer's description

### Quantification of RNA Using a Qubit Fluorometer

Total RNA was measured using the Qubit RNA HS Assay Kit (ThermoFisher, # Q32852) and a Qubit Flex system according to the manufacturer's description.

### Bioanalyzer Quality Control

Prior to labeling, the quality of RNA preparations was validated using an Agilent 2100 Bioanalyzer electrophoretic chip system (Agilent small RNA kit, #5067‐1548) according to the manufacturer's description. In brief, the isolated total RNA was diluted to 1–100 ng µL^–1^. After preparing the gel matrix, 2 µL of dye concentrate was added and the gel‐dye mix was loaded into the small RNA‐chip using the Agilent chip priming station. One microliter of exosomal RNA was analyzed using Agilent 2100 Bioanalyzer and the Agilent 2100 Expert software.

### miRNA Microarray

miRNA expression analysis was carried out using a SurePrint G3 Human miRNA Microarray Release 21.0 (Design ID 07 0156, Agilent Technologies). Samples were processed with the miRNA Complete Labeling & Hybridization Kit (Agilent Technologies, Santa Clara, CA, USA) according to the manufacturer's guidelines. Slides were scanned using a G2565CA microarray scanner (Agilent Technologies, Santa Clara, CA, USA). Expression data were extracted using the Feature Extraction software (Agilent Technologies). Preprocessing of expression data was performed according to Agilent's standard workflow. Using 5 quality flags (gIsPosAndSignif, gIsFeatNonUnifOL, gIsWellAboveBG, gIsSaturated, and gIsFeatPopnOL) from the Feature Extraction software output, probes were labeled as detected, not detected, or compromised. Gene expression levels were background corrected, and signals for duplicated probes were summarized by the geometric mean of noncompromised probes. After log_2_‐transformation, a percentile shift normalization at the 75% level was performed.

### Mass Spectrometry (MS)

MS was performed on sEVs derived from PTC and vehicle‐treated HUVECs were isolated after 24 h (*n* = 5 samples per condition). Additionally, MS was done on mouse total lung lysates of the following groups: sham+vehicle, sham+GW4869, TxT+vehicle, and TxT4869 (*n* = 5 samples per group).

*Sample Preparation*: Enriched sEVs were dissolved in lysis buffer (7 m Urea, 2 m Thiourea, 30 × 10 ^–3^ m Tris pH 8.5). Following reduction with DTT (5 × 10 ^–3^ m, AppliChem, Darmstadt, Germany) for 20 min at RT and subsequent alkylation with iodoacetamide (Sigma–Aldrich, St. Louis, USA) for 20 min at 37°C, samples were diluted with ammonium bicarbonate (50 × 10 ^–3^ m) at a ratio of 1:6. Trypsin was added in a 1:50 enzyme–protein ratio and digested overnight at 37 °C. Peptides were vacuum dried and dissolved in 15 µL TFA (0.1%). *MS Analysis*: Employing an LTQ Orbitrap Velos Pro system (Thermo Fisher Scientific, Bremen, Germany) online coupled to a U3000 RSLCnano (Thermo Fisher Scientific, Idstein, Germany), samples were analyzed as described previously,^[^ ^82^ ^]^ with the exception of prolonging the peptide elution gradient to 150min. *MS Data Analysis and Statistics*: Database search was performed using MaxQuant Ver. 1.6.3.4 (www.maxquant.org).^[^ ^83^ ^]^ Employing the built‐in Andromeda search engine, MS/MS spectra were correlated with the UniProt human or mouse reference proteome set (www.uniprot.org) for peptide identification. Carbamidomethylated cysteine was considered as a fixed modification along with oxidation (M), and acetylated protein N‐termini as variable modifications. False Discovery rates were set on both peptide and protein levels to 0.01. To assess exosomal enrichment, Uniprot was queried for the GOs extracellular exosome [70 062], exosome (RNase complex) [00178], and cytoplasmic exosome (RNase complex) [00177]. Retrieved protein IDs were compared to the set of identified proteins. For statistical analysis, Student's *t*‐test was performed on LFQ intensities, proteins having *P*‐values < 0.05 and absolute log_2_‐values > 2 were considered significantly regulated.

### RNA Sequencing

RNA sequencing was commercially done in the Biomedical Sequencing Facility in Vienna, Austria on mouse total RNA from lung lysates of the following groups: sham+vehicle, sham+GW4869, TxT+vehicle, and TxT4869 (*n* = 5 samples per group). Additionally, RNA‐Sequencing was performed on HUVECs treated for 8 h with patient‐derived sEVs sampled 4 and 24 h after hospital admittance (*n* = 6 samples per condition) or for 8 h with healthy control sEVs. *NGS Library Preparation*: The amount of total RNA was quantified using the Qubit 2.0 Fluorometric Quantitation system (Thermo Fisher Scientific, Waltham, MA, USA) and the RNA integrity number (RIN) was determined using the Experion Automated Electrophoresis System (Bio‐Rad, Hercules, CA, USA). RNA‐seq libraries were prepared with the TruSeq Stranded mRNA LT sample preparation kit (Illumina, San Diego, CA, USA) using Sciclone and Zephyr liquid handling workstations (PerkinElmer, Waltham, MA, USA) for pre‐ and post‐PCR steps, respectively. Library concentrations were quantified with the Qubit 2.0 Fluorometric Quantitation system (Life Technologies, Carlsbad, CA, USA) and the size distribution was assessed using the Experion Automated Electrophoresis System (Bio‐Rad, Hercules, CA, USA). For sequencing, samples were diluted and pooled into NGS libraries in equimolar amounts. *Sequencing and Raw Data Processing*: Expression profiling libraries were sequenced on HiSeq 3000/4000 instruments (Illumina, San Diego, CA, USA) in 50‐base‐pair, single‐end mode. Base calls, provided by the real‐time analysis (RTA) software (Illumina, San Diego, CA, USA), were subsequently converted into multiplexed, unaligned BAM format before demultiplexing into sample‐specific, unaligned BAM files. For raw data processing of the instruments, custom programs, based on Picard tools (https://broadinstitute.github.io/picard/), were used. *Transcriptome Analysis (STAR Aligner and DESeq2)*: NGS reads were mapped to the Genome Reference Consortium GRCm38 assembly via “Spliced Transcripts Alignment to a Reference” (STAR) using the “basic” Ensembl transcript annotation from version e99 (January 2020) as reference transcriptome. Because the mm10 assembly flavor of the UCSC Genome Browser was preferred for downstream data processing with Bioconductor packages for entirely technical reasons, Ensembl transcript annotation had to be adjusted to UCSC Genome Browser sequence region names. STAR was run with options suggested by the ENCODE project. Aligned NGS reads overlapping Ensembl transcript features were counted with the Bioconductor GenomicAlignments::summarizeOverlaps() function, taking into account that the Illumina TruSeq stranded mRNA protocol leads to the sequencing of the second strand so that all reads needed inverting before counting. Transcript‐level counts were aggregated to gene‐level counts and the Bioconductor DESeq2 package was used to test for differential expression based on a model using the negative binomial distribution.

### Enrichment Analysis Using EnrichR

Unweighted enrichment analyses for RNAseq and MS data were performed using the EnrichR meta‐analysis web tool (https://amp.pharm.mssm.edu/Enrichr/). For enrichment analysis of RNA‐sequencing data from mouse lungs, the following conditions were analyzed: TxT(+vehicle), TxT(+GW4869), and sham(+GW4869) versus sham(+vehicle). Subsequently, all significantly regulated transcripts with a cutoff of (log 2≥ +2, ≤‐2) were used for EnrichR analysis, and the first 20, significantly enriched terms were analyzed for signatures associated with trauma‐relevant processes in the TxT(+vehicle) versus sham(+vehicle) sample, except for the Exosome‐related signatures in Jensen's Compartment database that were listed in the first 30 significantly regulated terms. The identified terms were presented according to percent overlap with signatures, as well as with ‐log10 (adjusted *P*‐values). Enriched terms were looked up in the other conditions to evaluate whether GW4869 inhibition would impair percent overlap or significance. Grouping of the respective terms into the indicated functional sub‐groups for statistical analysis of mean percent overlap with signatures is shown in Supporting Information Data File 3. For MS of mouse lungs, similar conditions were investigated. To this end, all significantly up‐ or downregulated genes were used without cutoff and all significantly enriched terms were subsequently analyzed manually for signatures associated with the regulation of trauma‐relevant processes in the TxT(+vehicle) versus sham(+vehicle) sample. The identified terms were presented as described above. Grouping into functional sub‐groups for statistical analysis of mean percent overlap is also shown in Supporting Information Data File 8. RNA‐sequencing was further conducted on samples isolated from HUVEC cells treated with healthy control (HC) or polytrauma‐patient (PT) sEVs obtained 4 and 24 h upon admittance to the ER. Due to higher noise and variances in RNAseq data, low cutoff values (log_2_ ≥ +0.135, ≤ ‐0.135) for significantly regulated transcripts were used during enrichment meta‐analyses. The following conditions were evaluated: PT (4 h) versus HS and PT (24 h) versus HS. All significantly enriched terms were subsequently evaluated manually for signatures associated with the regulation of trauma‐relevant processes. Principal component analysis (PCA) using PC1 and PC2 as well as unsupervised hierarchical clustering were performed by the ClustVis online tool (https://biit.cs.ut.ee/clustvis/).^[^ ^84^ ^]^ Prediction ellipses in PCA scatter blots are drawn according to the 0.95 confidence level.

### Medical Art Illustrations

Illustrations were created using Servier medical art templates (https://smart.servier.com/) with minor modifications according to terms of the creative commons attribution 3.0 license agreement (https://creativecommons.org/licenses/by/3.0/).

### Quantification and Statistical Analysis

Quantification of Western blot bands was conducted using ImageJ “Gels Submenu” or “Gel plotting macros” (https://imagej.nih.gov/nih‐image/manual/tech.html#analyze). Both use a simple graphical method that involves generating lane profile plots, drawing lines to enclose peaks of interest, and then measuring peak areas using the wand tool.

Statistical analysis was performed using Prism software, version 8.42, for Windows (GraphPad, San Diego, CA). Graphs depict mean ± SEM for all conditions. Statistical significance: ns, not significant, *P = 0.05–0.01, **P = 0.01–0.001, ***P < 0.001, ****P < 0.0001.

## Conflict of Interest

The authors declare no conflict of interest.

## Authors Contributions

T.E. and Thomas Seufferlein conceived the project. T.E., Thomas Seufferlein, and S.P. designed and supervised the experiments. T.E., Thomas Seufferlein, and Tanja Seibold interpreted results and wrote the paper with input from all authors. Tanja Seibold, J.S., M.A., and A.P. performed TxT animal experiments. M.H.L. and P.R. provided blood from mouse and pig HS experiments. A.L. performed microarray analysis. Tanja Seibold, J.S., F.W., C.W., M.W., S.P., R.H., E.K., and T.E. performed in vitro experiments. M.H.L. and M.K. performed the polytrauma study in patients.

## Supporting information

Supporting Information

Supplemental Table 1

Supplemental Table 2

Supplemental Data 1

Supplemental Data 2

Supplemental Data 3

Supplemental Data 4

Supplemental Data 5

Supplemental Data 6

Supplemental Data 7

Supplemental Data 8

Supplemental Data 9

Supplemental Data 10

Supplemental Data 11

Supplemental Data 12

## Data Availability

Data available on request from the authors
