# Supplementary material for: Small Extracellular Vesicles Propagate the Inflammatory Response After Trauma
Source: Adv Sci (Weinh). 2021 Oct 28;8(24):2102381. doi: 10.1002/advs.202102381 (PMC8693079; doi:10.1002/advs.202102381)

© 2021 Wiley-VCH GmbH

ADVANCED SCIENCE
Open Access

Supporting Information

for *Adv. Sci.,* DOI: 10.1002/advs.202102381

Small Extracellular Vesicles Propagate the Inflammatory Response After Trauma

Tanja Seibold, Jonathan Schönfelder, Florian Weeber, André Lechel, Milena Armacki, Mareike Waldenmaier, Christoph Wille, Annette Palmer, Rebecca Halbgebauer, Ebru Karasu, Markus Huber-Lang, Miriam Kalbitz, Peter Radermacher, Stephan Paschke, Thomas Seufferlein, and Tim Eiseler**

Supplemental information

Figure S1: *In-vivo* and *in-vitro* trauma induce EV secretion. (a) Representative NTA curves of sEVs isolated from serum of sham and TxT mice 4hrs after trauma. **(b)** Kinetic of transendothelial electrical resistance (TEER) for confluent HUVECs on transwell filters after treatment with vehicle, PTC or thrombin. **(c)** qPCR of relative transcript levels in HUVECs treated with PTC for 24hrs. **(d)** Protein levels in TCL of HUVECs treated with PTC for 24hrs. Graphs show relative integrated densities normalized to actin and vehicle treatment. **(e)** CellTiterGlow2 viability assay of HUVEC cells treated with PTC and vehicle under serum-free conditions for 24hrs, as used for sEV-secretion experiments. **(f)** Representative NTA curves of HUVEC sEVs. **(g)** Total protein and total RNA levels in vehicle and PTC sEVs normalized to particle numbers determined by NTA. **(h)** sEV-uptake assay with the indicated amounts of ExoGlow-labled sEVs for 4hrs. Uptake was measured by flow cytometry (FCM). **(i)** qPCR of relative transcript levels in HUVECs treated with vehicle- or PTC- sEVs and supernatants (SN) for 8hrs. Purified sEV-samples were pelleted at 100,000 x g for 2hrs at 4°C in an ultracentrifuge The fractionated SNs and resuspended pellets were subsequently used to treat HUVEC cells and transcript levels were determined using qPCR. Transcript levels are normalized to vehicle sEV treatments. **(j)** Dose-dependent adhesion of primary CellTracker DeepRed labelled human PMNs to HUVEC cells after pre-treatment with the indicated amounts of sEVs for 8hrs. **(k)** Western blots of samples after *in-vitro* translation of RNA isolated from purified vehicle- and PTC-sEV. One of three independent experiments is shown. Statistical tests: (c, e, g, j) Two-tailed unpaired students t-test; (d) Ratio paired t-test. (i) Ordinary one-way ANOVA with Tukey's multiple comparison test. N-numbers indicate the number of independent samples. * $p < 0.05$; ** $p < 0.01$; ***$p < 0.001$, ****$p < 0.0001$; ns: no significant difference.

Figure S2: GW4869 reverses whole transcriptome TxT signatures in mouse lungs. (a) Cross-reference of all significant differentially regulated proteins detected in PTC-sEVs vs. vehicle-sEVs MS data **(Fig. 3h)** with the terms "cytoplasm" and "extracellular vesicular exosome" found in GO 2021 database**. (b)** Comparison of differentially regulated proteins

detected in MS data of PTC-sEVs vs. vehicle-sEVs associated with the term “exosome” in the Vesiclepedia database. **(c)** WB of MVP levels in vehicle and PTC-derived sEVs. **(d)** Unweighted EnrichR meta-analysis identified 171 significantly regulated trauma-associated terms in the TxT+vehicle vs. sham+vehicle RNAseq dataset (log2≥+2, ≤-2; first 20 significant enriched terms selected). The graph depicts percent overlap for enriched terms in the indicated conditions and -log10-p-values. Right-hand side: Numbers indicate significantly regulated transcripts for the different conditions during RNAseq with and without the indicated threshold levels. **(e)** Subgroup analyses of enriched terms found in **d**, grouped according to physiological or molecular parameters. **(f)** Subgroup analyses of enriched terms associated with exosomes in Jensen´s compartments found in **d**. N-numbers indicate the number of enriched terms in subgroups. Statistical tests: (c) Ratio paired t-test; (e, f) Ordinary one-way ANOVA with Tukey’s multiple comparison test. * $p < 0.05$; ** $p < 0.01$; ***$p < 0.001$, ****$p < 0.0001$; ns: no significant difference.

Figure S3: GW4869 reverses TxT proteome signatures in mouse lungs. (a) Left-hand side: Enriched trauma-associated terms found in the TxT+vehicle vs sham+vehicle MS data by unweighted EnrichR meta-analysis of all significantly upregulated proteins identified 26 significant signatures. The graph depicts percent overlap for enriched terms in the indicated conditions and -log10-p-values. Middle panel: Trauma-associated terms found in the TxT+vehicle vs. sham+vehicle MS data for all significantly downregulated proteins identified 45 significant signatures. The graph depicts percent overlap and -log10-p-values. Right-hand side: Numbers indicate significantly regulated proteins during MS for the different conditions. **(b)** Subgroup analyses of enriched terms found in **a**, grouped according to the indicated phenotypical or molecular parameters. N-numbers indicate the number of enriched terms in subgroups. **(c)** Multiplex-ELISA of the indicated cytokines in lung lysates of TxT mice and sham controls. N-numbers indicate the number of independent samples. Statistical test: (c) Ordinary one-way ANOVA with Holm Sidak multiple comparison test. * $p < 0.05$; ** $p < 0.01$; ***$p < 0.001$, ****$p < 0.0001$; ns: no significant difference.

Figure S4: Gene expression in mouse lungs after injection of sham and TxT-sEVs (a) Full Multiplex-ELISA of cytokines in the plasma of TxT mice and sham controls partially shown in **Fig. 4a**. **(b)** qPCR of relative transcript levels for inflammation- and endothelial-relevant genes in mouse lungs 4 and 16hrs after re-injection of sham- and TxT-sEVs. mRNA levels were normalized to the sham animals at corresponding time points. Data is shown as heatmap. **(c)** qPCR for relative transcript levels of CAMs in HUVECs treated as indicated for 24 hrs with PTC and GW4869 (10 µM). mRNA levels were normalized to DMSO treated cells. N-numbers indicate the number of independent samples. Statistical tests: (b, c) Ordinary one-way ANOVA with Holm Sidak multiple comparison test. * $p < 0.05$; ** $p < 0.01$; ***$p < 0.001$, ****$p < 0.0001$; ns: no significant difference.

Supplemental data files

Supplemental table 1

Patient and proband data

Supplemental table 2

Key resource materials

Supplemental data 1

MS in PTC-sEVs vs. vehicle-sEVs. All detected proteins.

Supplemental data 2

miRNA micro array in PTC-sEVs vs. vehicle-sEVs. Significant up- and downregulated miRNAs.

Supplemental data 3

RNASeq in murine lungs. EnrichR enriched terms between indicated groups (gene list cutoff $\log2 \geq +2, \leq -2$).

Supplemental data 4

Significant differentially regulated genes TxT+GW4869 vs. sham+vehicle.

Supplemental data 5

Significant differentially regulated genes TxT+vehicle vs. sham+vehicle.

Supplemental data 6

Significant differentially regulated genes sham+GW4869 vs. sham+vehicle

Supplemental data 7

MS in murine lungs. All detected proteins.

Supplemental data 8

MS in murine lungs. EnrichR enriched terms between indicated groups (protein list cutoff: all significantly regulated proteins).

Supplemental data 9

36-plex multiplex ELISA from murine plasma samples.

Supplemental data 10

RNASeq in HUVECs treated with patient derives sEVs. EnrichR enriched terms between indicated groups (gene list cutoff log2≥+0.135, ≤-0.135)

Supplemental data 11

Significant regulated genes in HUVECs treated with patient derived sEVs (4hrs after trauma) vs. HC.

Supplemental data 12

Significant regulated genes in HUVECs treated with patient derived sEVs (24hrs after trauma) vs. HC.

Supplemental Figure 1

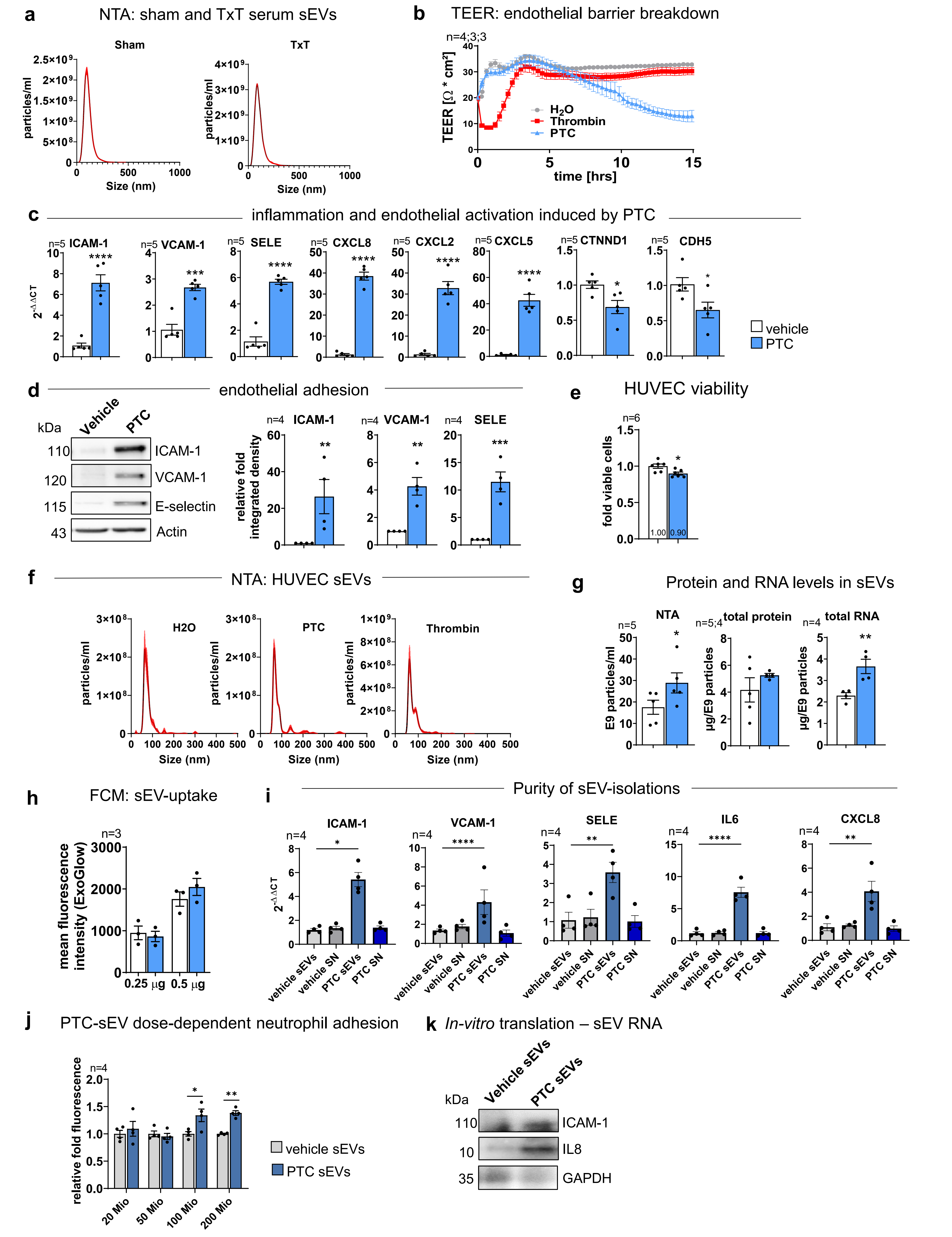

Supplemental Figure 2

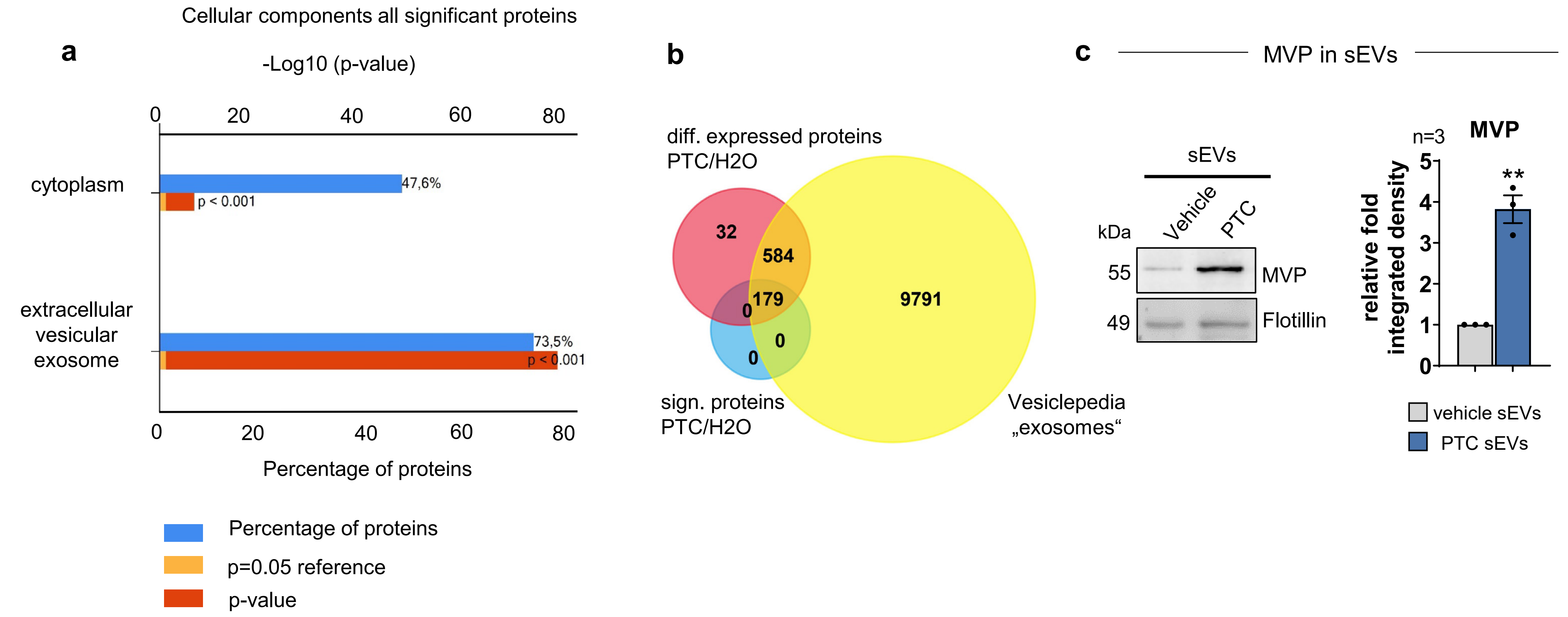

Mouse lungs: TxT +/- GW4869 (RNAseq)

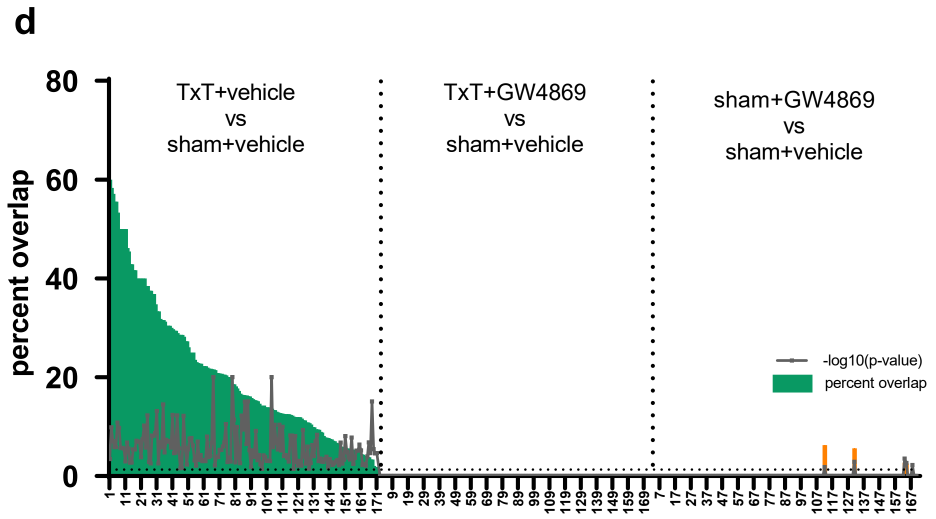

Group (RNASeq)	significant up (w/o cutoff)	significant down (w/o cutoff)	significant up (cutoff log2≥2)	significant down (cutoff log2≤-2)
**TxT+vehicle vs sham+vehicle**	3337	3231	144	52
**TxT+GW4869 vs sham+vehicle**	1251	1224	0	2
**sham+GW4869 vs sham+vehicle**	38	36	25	10

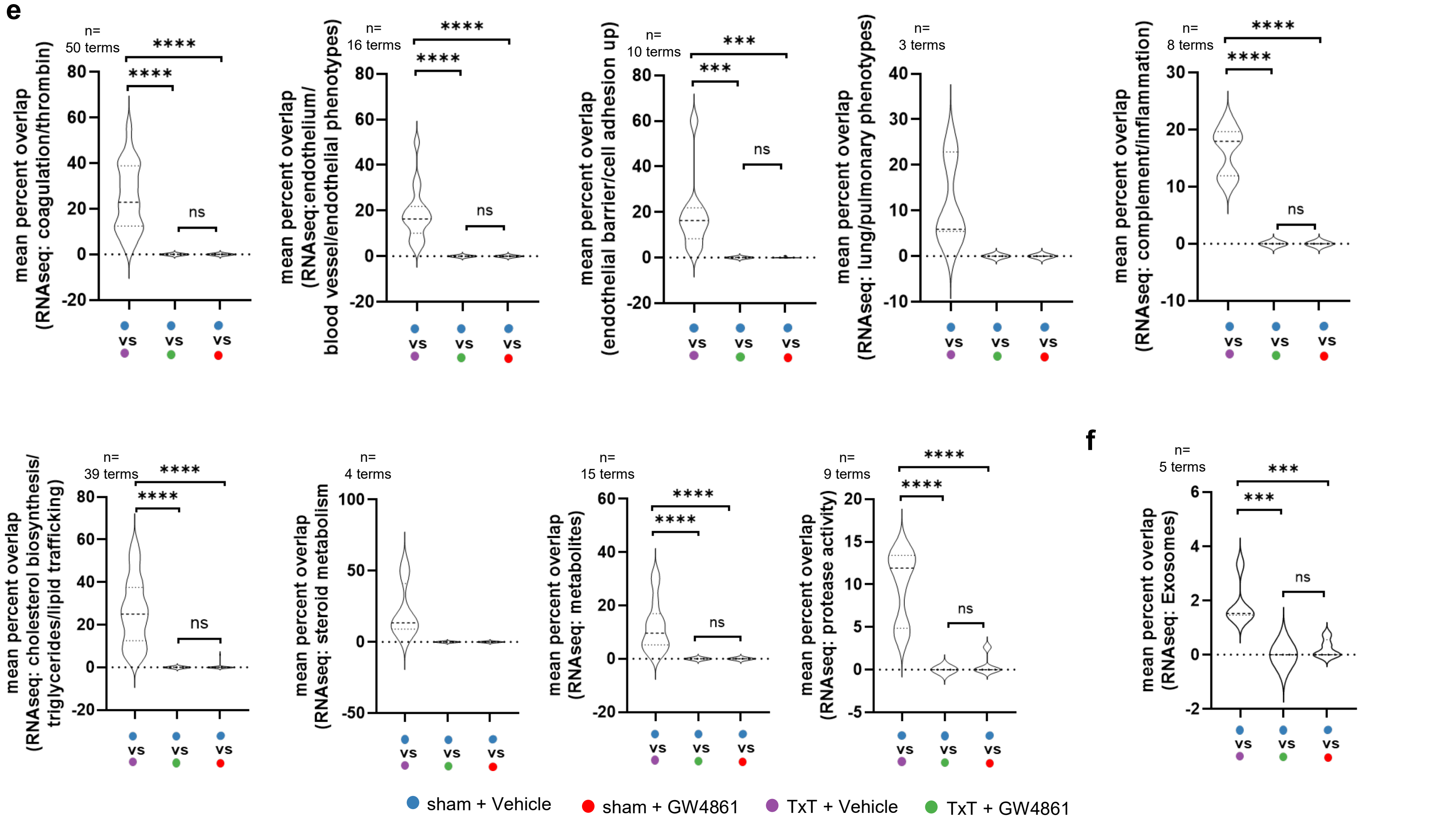

Supplemental Figure 3

MS: Mouse lungs: TxT +/- GW4869

a

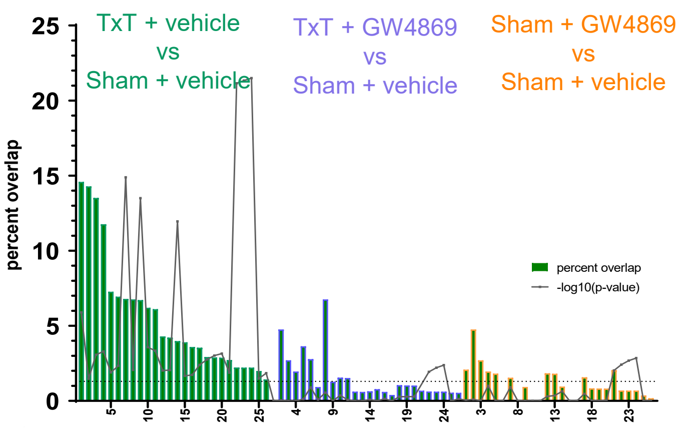

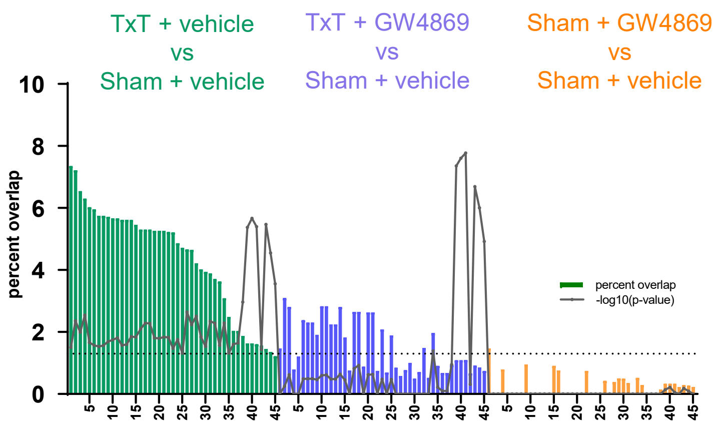

Group (MS)	significant up	significant down
TxT+vehicle vs sham+vehicle	109	134
TxT+GW4869 vs sham+vehicle	39	60
sham+GW4869 vs sham+vehicle	44	22

1 Cortical actin cytoskeleton (JC)
2 RHO GTPases activate PAKs Homo sapiens R-HSA-5627123 (R 2016)
3 Apoptotic cleavage of cellular proteins Homo sapiens R-HSA-111465 (R 2016)
4 Apoptotic execution phase Homo sapiens R-HSA-75153 (R 2016)
5 actin filament (GO:0005884; CC 2018)
6 Adherens junction (KEGG 2019M)
7 cell-cell adherens junction (JC)
8 Adherens junction cell adhesion (BP 2019)
9 cadherin binding (GO:0045296; MF 2018)
10 Cell-cell communication (BP 2019)
11 Cell-Cell communication Homo sapiens R-HSA-1500931 (R 2016)
12 Apoptosis Homo sapiens R-HSA-109581 (R 2016)
13 Programmed Cell Death Homo sapiens R-HSA-5357801 (R 2016)
14 cell-cell junction (JC)
15 Apical junction complex (JC)
16 Tight junction (KEGG 2019M)
17 actin binding (GO:0003779; MF 2018)
18 neutrophil degranulation (GO:0043312; BP 2018)
19 neutrophil activation involved in immune response (GO:0002283; BP 2018)
20 neutrophil mediated immunity (GO:0002446; BP 2018)
21 actin cytoskeleton (GO:0015629; CC 2018)
22 Extracellular exosome (JC)
23 Extracellular vesicle (JC)
24 Extracellular organelle (JC)
25 Hemostasis Homo sapiens R-HSA-109582 (R 2016)
26 Endomembrane system (JC)

1 Messenger RNA splicing: major pathway (BP 2019)
2 protein targeting to ER (GO:0045047; BP 2018)
3 SRP-dependent cotranslational protein targeting to membrane Homo sapiens R-HSA-1799339 (R 201
4 Spliceosome (BP 2019)
5 ECM-receptor interaction (KEGG 2019M)
6 Peptide chain elongation Homo sapiens R-HSA-156902 (R 2016)
7 Selenocysteine synthesis Homo sapiens R-HSA-2408557 (R 2016)
8 Eukaryotic Translation Termination Homo sapiens R-HSA-72764 (R 2016)
9 Platelet degranulation Homo sapiens R-HSA-114608 (R 2016)
10 L13a-mediated translational silencing of Ceruloplasmin expression Homo sapiens R-HSA-156827 (R 2
11 3' -UTR-mediated translational regulation Homo sapiens R-HSA-157279 (R 2016)
12 Eukaryotic Translation Elongation Homo sapiens R-HSA-156842 (R 2016)
13 Nonsense Mediated Decay (NMD) independent of the Exon Junction Complex (EJC) Homo sapiens R-
14 GTP hydrolysis and joining of the 60S ribosomal subunit Homo sapiens R-HSA-72706 (R 2016)
15 Response to elevated platelet cytosolic Ca2+ Homo sapiens R-HSA-76005 (R 2016)
16 Spliceosome (KEGG 2019M)
17 Translation (BP 2019)
18 Translation Homo sapiens R-HSA-72766 (R 2016)
19 spliceosomal complex (GO:0005681; CC 2018)
20 Cap-dependent Translation Initiation Homo sapiens R-HSA-72737 (R 2016)
21 Eukaryotic Translation Initiation Homo sapiens R-HSA-72613 (R 2016)
22 mRNA Splicing - Major Pathway Homo sapiens R-HSA-72163 (R 2016)
23 Formation of a pool of free 40S subunits Homo sapiens R-HSA-72689 (R 2016)
24 mRNA Splicing Homo sapiens R-HSA-72172 (R 2016)
25 Nonsense Mediated Decay (NMD) enhanced by the Exon Junction Complex
26 RNA splicing, via transesterification reactions with bulged adenosine as nucleophile
27 Spliceosomal complex (JC)
28 mRNA splicing, via spliceosome (GO:0000398; BP 2018)
29 Protein complex involved in cell-matrix adhesion (JC)
30 Messenger RNA processing (BP 2019)
31 mRNA processing (GO:0006397; BP 2018)
32 endoplasmic reticulum lumen (GO:0005788; CC 2018)
33 Processing of Capped Intron-Containing Pre-mRNA Homo sapiens R-HSA-72203
34 focal adhesion (GO:0005925; CC 2018)
35 Protein metabolism (BP 2019)
36 Intracellular ribonucleoprotein complex (JC)
37 Ribonucleoprotein complex (JC)
38 RNA binding (GO:0003723; MF 2018)
39 Extracellular vesicle (JC)
40 Extracellular organelle (JC)
41 Extracellular exosome (JC)
42 Extracellular space (JC)
43 membrane-bounded vesicle (JC)
44 Extracellular region part (JC)
45 Extracellular region (JC)

b

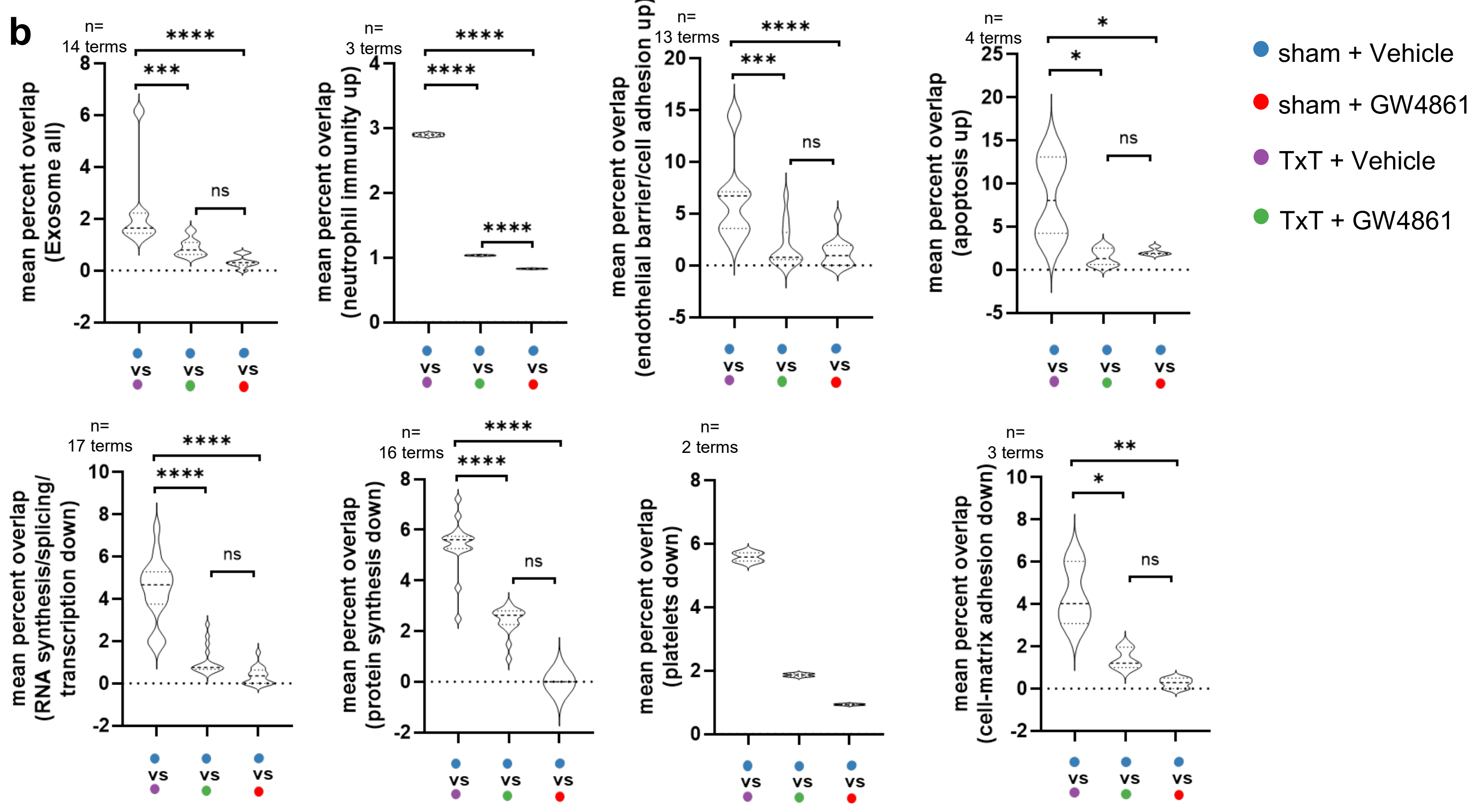

c mouse lungs: multiplex-ELISA

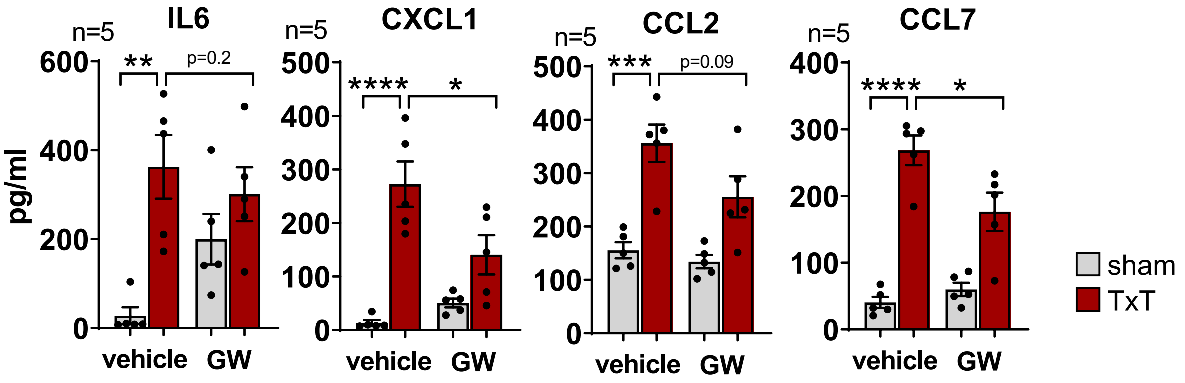

Supplemental Figure 4

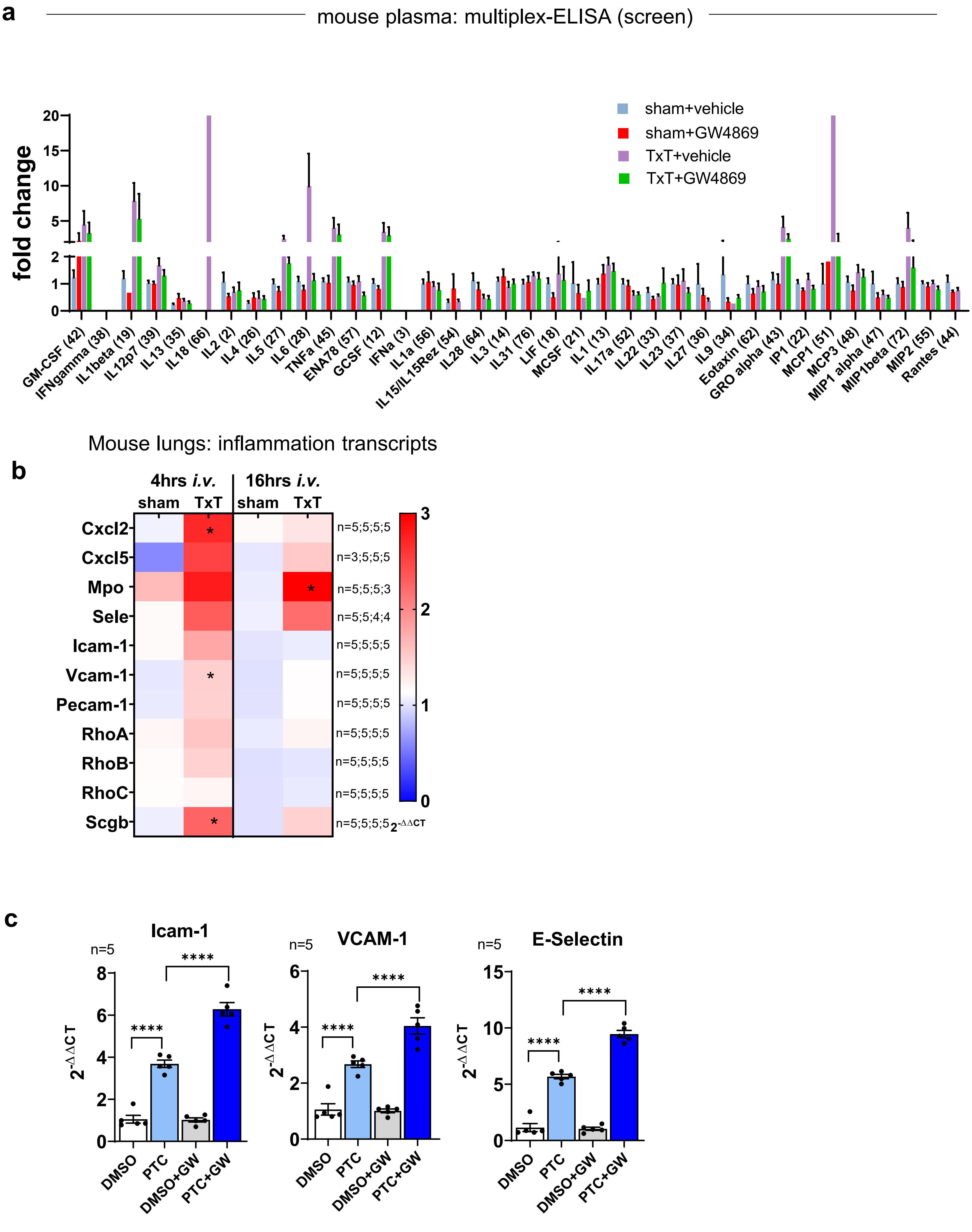

Supplement: Supplementary file 1 — Supporting Information [file ADVS-8-2102381-s013.pdf]
