# Supplementary material for: Small Extracellular Vesicles Propagate the Inflammatory Response After Trauma
Source: Adv Sci (Weinh). 2021 Oct 28;8(24):2102381. doi: 10.1002/advs.202102381 (PMC8693079; doi:10.1002/advs.202102381)
Supplement: Supplementary file 2 — Supplemental Table 1 [file ADVS-8-2102381-s004.pdf]

Supplemental table 2: Polytrauma patient and proband data

patient	sex	age	ISS	initial IL-6 (pg/ml)	initial CRP	initial Hematokrit (L/L)	initial Hb (g/dl)
PT1	m	47	38	225,4	1,7	0,37	13,1
PT2	m	40	75				
PT3	m	63	43	2,2	0,8	0,41	14,1
PT4	m	21	41	274,9	0,29	0,35	12
PT5	m	33	29	373,8	6	0,36	11,6
PT6	m	52	43	245,6	2,9	0,43	14,3
**proband**							
HC1	w	27					
HC2	w	27					
HC3	m	27					
HC4	m	54					
HC5	w	27					
HC6	m	28					
HC7	w	58					
HC8	w	31					
HC9	w	30					
