# Supplementary material for: Small Extracellular Vesicles Propagate the Inflammatory Response After Trauma
Source: Adv Sci (Weinh). 2021 Oct 28;8(24):2102381. doi: 10.1002/advs.202102381 (PMC8693079; doi:10.1002/advs.202102381)
Supplement: Supplementary file 3 — Supplemental Table 2 [file ADVS-8-2102381-s002.pdf]

KEY RESOURCES

REAGENT or RESOURCE	SOURCE	IDENTIFIER
**Antibodies**		
anti-β-actin AC-15	Sigma-Aldrich	A1978
anti-GRP94	ThermoFisher Scientific	PA5-23177
anti TSG-101	GeneTex Inc.	GTX70255
anti-β-Catenin	BD Transduction Laboratories	610154
anti-E-Selectin	SantaCruz	sc-137054
Anti-IL8	SantaCruz	sc-376750
anti-ICAM-1	Abcam	ab179707
anti-Myeloperoxidase	Abcam	ab9535
anti-p120-catenin	SantaCruz	sc-13957
anti-RhoA	Cell Signaling	2117S
anti-RhoB	SantaCruz	sc-8048
anti-Rab 27a	SantaCruz	sc-136996
anti-Rab11	Abcam	ab3612
anti-RAb31	Abcam	ab224421
anti-SP-D	SantaCruz	sc-59695
anti-VCAM-1	SantaCruz	sc-1316
anti-VE-Cadherin	SantaCruz	sc-28644
anti-CD63	Santa Cruz	sc-5275
anti-Rab11a	Santa Cruz	sc-166912
anti-Rab31	Abcam	ab224421
anti-Flotillin	Cell Signalling	3253S
anti-CD81	SantaCruz	sc-7637
anti-WDR1	SantaCruz	sc-393159
anti-RPS3	SantaCruz	sc-376008
anti-EEF2	SantaCruz	sc-166415
anti-mouse IgG HRP	GE Healthcare Life Sciences	NA931V

anti-rabbit IgG HRP	GE Healthcare Life Sciences	NA934V
Alexa-Fluor 488/568/647 goat-anti-mouse	ThermoFisher Scientific	#A11031<br>#A11001<br>#A21235
Alexa-Fluor 488/568/647 goat-anti-rabbit	ThermoFisher Scientific	# A11034<br># A11011<br># A21244
Alexa-Fluor 647 Phalloidin	ThermoFisher Scientific	A22287
**Chemicals, Peptides, and Recombinant Proteins**		
Thrombin from human plasma	Sigma Aldrich	T6884
GW4869	Sigma	D1692-5MG
**Components PTC**		
IL1ß (200 pg/ml)	PeproTech	200-01B
IL6 (500 pg/ml)	PeproTech	200-06
CXCL8 (150 pg/ml)	PeproTech	200-08
C3a (500 ng/ml)	abbexa	abx066057
C5a des-arg (10 ng/ml)	Merck	204902-30UG
**Components CovC**		
IL1ß (2 pg/ml)	PeproTech	200-01B
IL2 (8 pg/ml)	PeproTech	200-02
IL6 (25 pg/ml)	PeproTech	200-06
CXCL8 (20 pg/ml)	PeproTech	200-08
IL10 (10 pg/ml)	PeproTech	200-10
IFNy (10 pg/ml)	PeproTech	300-02
CCL2 (50 pg/ml)	PeproTech	300-04
CCL3 (5 pg/ml)	PeproTech	300-08
CCL4 (110 pg/ml)	PeproTech	300-09
TNFa (10 pg/ml)	PeproTech	300-01A
**Critical Commercial Assays**		

IL-6 human ELISA kit	ThermoFisher Scientific	BMS213-2
CXCL8 human ELISA	ThermoFisher Scientific	KHC0081
Mouse IL1beta ELISA Kit	R&D Systems	MHSL1300
Murine CD138 (Syndecan-1) ELISA kit	Diaclone	860.090.096
Mouse Lipocalin-2/NGAL ELISA	R&D Systems	MLCN20
ProcartaPlex mouse 36-plex	ThermoFisher Scientific	EPX360-26092-901
Procartaplex 10-plex custom	ThermoFisher Scientific	PPX-10
Urea assay	Sigma-Aldrich	MAK006
EZ-Magna RIP	Merck	17-701
Rabbit Reticulocyte Lysate System	Promega	L4960
**Deposited Data**		
Microarray and sequencing data	GSE172212	
**Experimental Models: Cell Lines**		
HUVEC	ATCC	CRL-1730
C57BL/6 primary lung microvascular endothelial cells	Pelo Biotech	PB-C57-6011
**Experimental Models: Organisms/Strains**		
C57BL/6	purchased from animal facility Ulm	
Bretoncelles Meishan (FBM) pigs	performed by co-operator Peter Radermacher	
**Mir-mimics/inhibitors**		
hsa-miR-298 *mir*Vana® miRNA mimic	ThermoFisher	4464066 MC12574
hsa-miR-34c-3p *mir*Vana® miRNA mimic	ThermoFisher	4464066 MC12245
mmu-miR-298-5p *mir*Vana® miRNA mimic	ThermoFisher	4464066 MC12525
mmu-miR-298-5p mirVana® miRNA inhibitor	ThermoFisher	4464084 MH12525
**Oligonucleotides**		
**Primer human**		
C3	Qiagen	QT00089698
C5aR-1	Qiagen	QT00997766
C5aR-1	Qiagen	QT00088011

CEACAM8	Qiagen	QT00000035
CCL2	Qiagen	QT00212730
CCR-1	Qiagen	QT00073549
CD44	Qiagen	QT00073549
CD99	Qiagen	QT00080661
CLDN5	Qiagen	QT00232197
CXCL1	Qiagen	QT00199752
CXCL2	Qiagen	QT00013104
CXCL5	Qiagen	QT00203686
CXCL8 (IL-8)	Qiagen	QT00000322
CXCR1	Qiagen	QT00212919
CXCR2	Qiagen	QT00000518
SELE (E-Selectin)	Qiagen	QT00015358
GAPDH	Qiagen	QT00079247
ICAM-1	Qiagen	QT00074900
IL1b	Qiagen	QT00021385
IL6	Qiagen	QT00083720
(CTNND1) p120-catenin	Qiagen	QT00033831
PECAM-1	Qiagen	QT00081172
SELP (P-Selectin	Qiagen	QT00012516
RHOB	Qiagen	QT00227409
RHOC	Qiagen	QT00083573
RPS3	Qiagen	QT00096859
SELPLG	Qiagen	QT00235963
CTNNB1 (ß-catenin)	Qiagen	QT00077882
TLR2	Qiagen	QT00236131
TNF-alpha	Qiagen	QT00029162
VCAM-1	Qiagen	QT00018347
CDH5 (VE-Cadherin)	Qiagen	QT00013244
WDR-1	Qiagen	QT00206409

miR-298	Qiagen	MS00009275
miR-34c-3p	Qiagen	MS00009548
**Primer murine**		
C3	Qiagen	QT00109270
Cc16	Qiagen	QT00105266
Ccl2	Qiagen	QT00167832
Cd68	Qiagen	QT00254051
Cldn5 (Claudin-5)	Qiagen	QT00254905
Cxcl1	Qiagen	QT00115647
Cxcl2	Qiagen	QT00113253
Cxcl5	Qiagen	QT01658146
Cxcr2	Qiagen	QT00283696
Sele (E-Selectin)	Qiagen	QT00114338
Gapdh	Qiagen	QT01658692
Gus9	Qiagen	QT00176715
Havcr1	Qiagen	QT00112427
Hmox1	Qiagen	QT00159915
Hpx	Qiagen	QT00098203
Icam-1	Qiagen	QT00155078
Il1ß	Qiagen	QT01048355
Il6	Qiagen	QT00098875
Klotho	Qiagen	QT00104293
Lcn2	Qiagen	QT00113407
Mpo	Qiagen	QT01065687
(Ctnnd1) p120-catenin	Qiagen	QT00146888
Pecam-1	Qiagen	QT01052044
Pnmt	Qiagen	QT00262815
Rab11a	Qiagen	QT00241906
Rab11b	Qiagen	QT00126959
Rab31	Qiagen	QT00016982

Rab7	Qiagen	QT01766912
RhoA	Qiagen	QT00197568
RhoB	Qiagen	QT00249648
RhoC	Qiagen	QT00103901
Spp1	Qiagen	QT02524536
Ctnnb1 (ß-catenin)	Qiagen	QT00160958
Tlr2	Qiagen	QT00236131
Tlr4	Qiagen	QT00259042
TNF-alpha	Qiagen	QT00104006
Vcam-1	Qiagen	QT00128793
(Cdh5) VE-Cadherin	Qiagen	QT00110467
miR-298-5p	Qiagen	MS00002016
miR-298-3p	Qiagen	MS00024696
miR-34a	Qiagen	MS00025697
miR-34b	Qiagen	MS00007910
miR-34c	Qiagen	MS00011907
**miR control primer**		
RNU6-2	Qiagen	MS00033740
SNORD61	Qiagen	MS00033705
SNORD96A	Qiagen	MS00033733
Other		
